# Elucidating the beneficial effects of gut microbiota-derived metabolites in treating hepatocellular carcinoma: an integrated network pharmacology, molecular docking, and single-cell sequencing analysis

**DOI:** 10.1186/s40643-026-01129-x

**Published:** 2026-09-12

**Authors:** Jinlin Huo, Wenqian Song, Yuan Sun, Jun Lu, Maolin Wang

**Affiliations:** 1 Clinical Medicine Research Center, The First Affiliated Hospital of Shantou University Medical College, Shantou, 515041 China; 2 State Key Laboratory of Southwestern Chinese Medicine Resources, School of Pharmacy, Chengdu University of Traditional Chinese Medicine, Chengdu, 610103 China

**Keywords:** Gut microbiota, Metabolites, Network pharmacology, Hepatocellular carcinoma, 3-indolepropionic acid

## Abstract

Hepatocellular carcinoma (HCC) is the most common type of liver cancer and the third leading cause of cancer-related mortality worldwide. While accumulating evidence has highlighted the beneficial effects of gut microbiota (GM)-derived metabolites in HCC treatment, the underlying mechanisms remain unclear. We retrieved GM-derived metabolites from gutMGene v2.0, acquired their targets using the Similarity Ensemble Approach (SEA) and Swiss Target Prediction (STP), and identified HCC-related targets from public databases. Overlapping targets were then obtained via Venn diagram analysis. We constructed protein–protein interaction (PPI) networks, analyzed signaling pathways, and established a comprehensive microbiota-metabolite-target-disease (M-M-T-D) network. We also examined the correlation between core gene expression and overall survival in HCC patients using UALCAN and GEPIA2. Single-cell transcriptomic data from TISCH2 were analyzed to explore the cellular distribution of core genes. To identify candidate compounds, we evaluated the ADMET properties of metabolites and determined the compound with the highest binding affinity via molecular docking. Ultimately, 8 core targets (AKT1, IL6, PPARG, IL1B, JUN, NFKB1, CASP3, and PTGS2) were identified. These targets interacted with seven core metabolites (10-oxo-12(Z)-octadecenoic acid, genipin, 3-indolepropionic acid, succinate, acetate, propionate, and butyrate) through the M-M-T-D network to exert inhibitory effects on HCC. Single-cell RNA sequencing (scRNA-seq) analysis revealed that core gene expression was correlated with the tumor microenvironment (TME) of HCC. Molecular docking confirmed that 3-indolepropionic acid exhibited the strongest binding affinity to the core targets. Collectively, GM-derived metabolites exert beneficial effects against HCC by regulating multiple signaling pathways and targets, supporting their potential as therapeutic candidates.

**Graphical abstract:**

**Figure:**
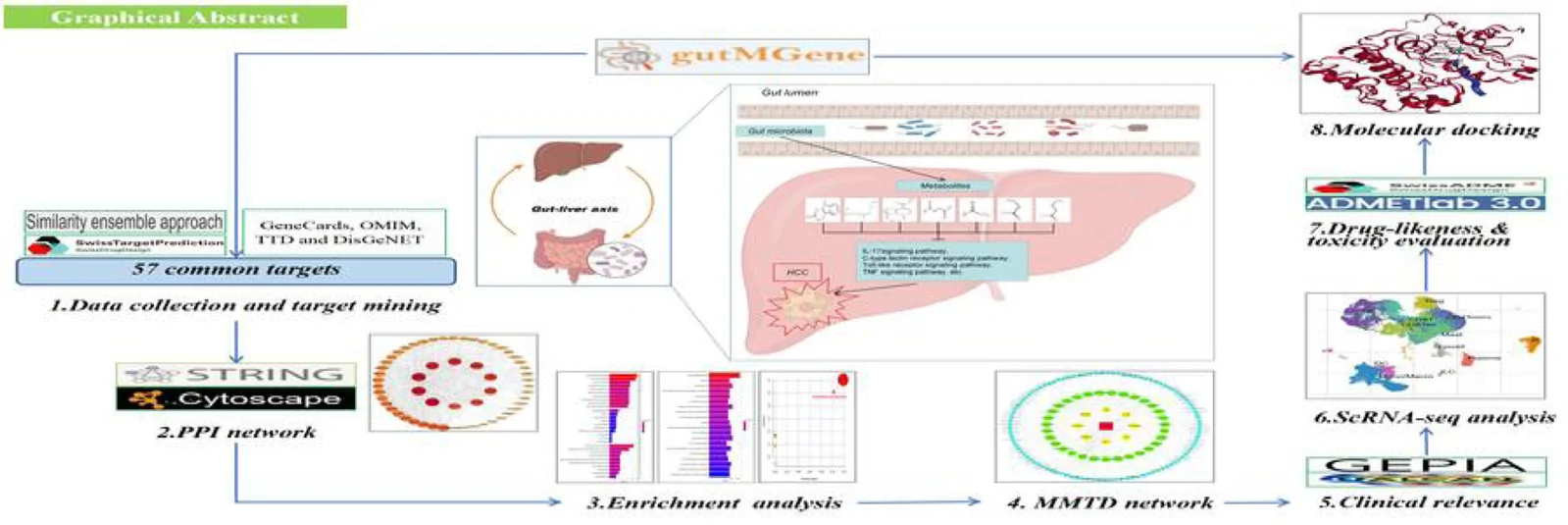

**Supplementary Information:**

The online version contains supplementary material available at https://doi.org/10.1186/s40643-026-01129-x.

## Introduction

Primary liver cancer (PLC) is a common malignant tumor and the third leading cause of cancer-related deaths worldwide (Yeo et al. 2025). Hepatocellular carcinoma (HCC) is the predominant histopathological type, accounting for approximately 90% of global PLC cases (European Association for the Study of the 2025). There are many treatment options for HCC, such as surgical resection, liver transplantation, chemotherapy, radiation therapy, tyrosine kinase inhibitors (TKIs), and immune checkpoint inhibitors (ICIs), but HCC patients still experience high recurrence and metastasis rates and poor survival (Chidambaranathan-Reghupaty et al. 2021). Therefore, it is urgent necessary to find many more effective methods to reduce its mortality.

Recent studies have highlighted that the gut microbiota (GM) plays a key role in regulating the course of liver diseases, especially by mediating related mechanisms through the production of microbial metabolites (such as bile acids [BAs] and short-chain fatty acids [SCFAs]) (Kang et al. 2022; Nie et al. 2024). Analysis of healthy individuals, cirrhotic patients, and HCC patients revealed a sequential decline in GM diversity, with the most pronounced reduction observed in patients with HCC (Ponziani et al. 2019). The GM and its metabolites interact closely with the liver through the "gut-liver axis", directly affecting the metabolism, immune and inflammatory responses of the liver, or indirectly regulating the pathophysiological processes of the liver by changing the intestinal barrier function, systemic metabolism and immune status (Hsu and Schnabl 2023; Schwabe and Greten 2020). Therefore, regulating the GM or its metabolites may be a potential prevention and treatment strategy for liver diseases (Yao et al. 2025). Recently, one report demonstrated that oral administration of complex probiotics containing *Bifidobacterium longum* after surgery in HCC patients can promote liver function recovery, shorten hospital stay and improve the one-year survival rate, and the relevant mechanism may be that *Bifidobacterium longum* reduces liver inflammation and fibrosis and inhibits liver cell proliferation by altering the metabolism of the bacteria, such as increasing serotonin, secondary bile acid and short-chain fatty acids (SCFAs) levels (Yu et al. 2024). Another report suggested that Bacteroides-derived acetic acid might modulate the immune microenvironment of HCC, promote cytotoxic CD8+ T cell function, and inhibit HCC development by affecting fatty acid metabolism and histone acetylation modification (Ma et al. 2024). The above studies indicate that there are potential relationships among HCC, the GM and metabolites. Although the beneficial effects of GM metabolites are well known, their specific pharmacological mechanisms in HCC have essentially remained obscure due to the complexity of metabolite-related targets and wide-ranging signaling pathways.

Network pharmacology offers a new view on the base of systems biology theory, biological system network analysis, and multi-target drug molecule design specific signal node selection (Hopkins 2007; Yao et al. 2026). Network pharmacology methods are currently recommended for interpreting drug–body interactions and guiding new drug discovery (Wang et al. 2025c; Zhao et al. 2023). Therefore, an increasing number of researchers are applying this method to discover new targets and design more effective, less toxic drugs (Nogales et al. 2022). In the present study, the metabolites, core targets, and signaling pathways of the GM -mediated HCC amelioration were investigated via network pharmacology, molecular docking, and single-cell sequencing analysis.

## Materials and methods

### Data collection and target mining

Detailed information on the data sources and tools were available in Table 1.

**Table 1:** Databases, software, and analysis platforms used in this study

No	Tool / Database	Version	Purpose in this study	URL
1	gutMGene	V2.0	Retrieval of GM-derived metabolites and their reported target genes	http://bio-computing.hrbmu.edu.cn/gutmgene/
2	Pubchem	–	Obtaining SMILES chemical structures of metabolites	https://pubchem.ncbi.nlm.nih.gov/
3	Similarity Ensemble Approach (SEA)	–	Prediction of potential protein targets for each metabolite	https://sea.bkslab.org/
4	Swiss Target Prediction(STP)	–	Prediction of potential protein targets for each metabolite	http://www.swisstargetprediction.ch/
5	Venny 2.1.0	–	Venn diagram analysis	https://bioinfogp.cnb.csic.es/tools/venny/
6	Genecards	–	Retrieval of HCC-related genes (Protein-coding genes; Relevance score ≥ 5)	https://www.genecards.org/
7	OMIM	–	Retrieval of HCC-related genes	https://omim.org/
8	DisgeNet	–	Retrieval of HCC-related genes	https://www.disgenet.org/
9	Therapeutic Target Database(TTD)	–	Retrieval of HCC-related genes	http://db.idrblab.net/ttd/
10	STRING	V12.0	Construction of protein–protein interaction (PPI) network	https://string-db.org/
11	Cytoscape software	V3.7.1	Visualization and topological analysis of PPI network, construction of M-M-T-D network	https://cytoscape.org/
12	MetaboAnalyst 6.0	V6.0	Metabolic pathway enrichment	https://www.metaboanalyst.ca/
13	UALCAN	–	Validation of the core target genes expression in HCC vs. normal tissues	http://ualcan.path.uab.edu
14	GEPIA2	V2.0	Survival analysis and expression validation of core target genes	http://gepia2.cancer-pku.cn/#index
15	TISCH2	V2.0	Tumor immune microenvironment analysis for target genes	http://tisch.comp-genomics.org/
16	CB-DOCK2	–	Molecular docking between metabolites and target proteins	https://cadd.labshare.cn/cb-dock2/
17	Protein Data Bank (PDB)	–	Retrieval of 3D structures of target proteins	https://www.rcsb.org
18	SwissADME	V2.0	Prediction of pharmacokinetic properties of metabolites	http://www.swissadme.ch/
19	ProTox 3.0	V3.0	Prediction of toxicity profiles of metabolites	https://tox.charite.de/protox3/
20	R4.4.0	V4.4.0	Statistical analysis and data visualization	https://www.r-project.org/

GM-derived metabolites and their known targets were first retrieved from the gutMGene v2.0 database (accessed on 15 March 2025), with records restricted to Homo sapiens to ensure physiological relevance. The Simplified Molecular Input Line Entry System (SMILES) strings for these metabolites were obtained from PubChem (accessed on 15 March 2025). For in silico target prediction, we employed two complementary platforms‒the Similarity Ensemble Approach (SEA) and SwissTargetPrediction (STP) (both accessed on 16 March 2025)—with searches restricted to human species. For SEA, targets with a *P*-value < 0.05 were retained as statistically significant; for STP, all targets with a probability score > 0 were kept to capture potential interactions with positive predictive likelihood. Finally, the overlapping targets between the two platforms were identified using Venny 2.1.0 and designated as high-confidence consensus targets for subsequent analyses (Yu et al. 2025).

Using "Hepatocellular Carcinoma" as the keyword, potential HCC-related genes were retrieved from the GeneCards, OMIM, TTD, and DisGeNET databases (all accessed on 16 March 2025). To ensure data reliability and specificity, the following filtering criteria were applied: for GeneCards, we included only protein-coding genes with a relevance score of at least 1. OMIM, TTD, and DisGeNET, as expert-curated resources without standardized numerical cutoffs, were directly incorporated without additional filtering. The resulting gene lists from all four databases were merged, and duplicates were removed to obtain a unified set of HCC-associated genes. The metabolite-predicted targets were then intersected with these HCC-related genes via Venn diagram analysis. Finally, the common targets were obtained by intersecting the above results with the targets retrieved from gutMGene v2.0.

### Protein–protein interaction (PPI) network construction and analysis

The common targets were mapped to the STRING (accessed on 19 March 2025) to identify their interactions, the organism was set to “*Homo sapiens*” mode, the minimum required interaction score was set to “medium confidence (0.400)”, disconnected nodes were hidden, and then the “tsv” file was downloaded to import the Cytoscape 3.7.1 to construct the PPI network (Shannon et al. 2003). To further mitigate the potential bias introduced by a single threshold, four complementary topological algorithms (Degree, MCC, MNC and Radiality) were employed to cross-validate the identified top 10 hub genes, ensuring that our findings are robust rather than threshold-dependent. The results from above four algorithms were intersected to obtain the final set of hub targets, which were considered potential core targets (Wang et al. 2025b).

To further address the influence of the interaction score threshold on network topology and hub genes selection, an additional sensitivity analysis was performed. Specifically, the PPI network was reconstructed using STRING (accessed on 19 March 2025) with a more stringent confidence threshold of 0.7. The same four topological algorithms (Degree, MCC, MNC, and Radiality) were applied to the 0.7-threshold network to identify core nodes and intersected to obtain the final set of core targets.

### Gene ontology(GO) functional and signaling pathway enrichment analyses

R packages (clusterProfiler, enrichplot, and ggplot2) were used for pathway enrichment analysis of gene functions. GO functional and Kyoto Encyclopedia of Genes and Genomes (KEGG) pathway analyses were utilized to annotate the functions of the hub targets. The GO terms includes three distinct categories: biological processes (BPs), cellular components (CCs), and molecular functions (MFs). The top 10 enriched GO terms and the top 20 KEGG signaling pathways were selected on based on their *P* values. The results were visualized using bar plots generated with the R package, based on the screening criteria of enrichment score,* P* value, and count values. The GM-derived metabolites corresponding to the targets were input into the MetaboAnalyst 6.0 (accessed on 19 March 2025) for the metabolic pathway enrichment analysis (Pang et al. 2024).

### Microbiota–metabolites–targets–disease (M-M-T-D) network diagram construction

Cytoscape 3.7.1 was used to create networks diagram to characterize the relationships among the microbiota, metabolites, core targets, and disease. The GM, metabolites, core targets, and disease were considered as nodes in the M-M-T-D network, and the relationships among these the four components were considered edges.

### Clinical relevance analysis

The UALCAN (Chandrashekar et al. 2022) (accessed on 20 March 2025) and GEPIA (Li et al. 2021a) (accessed on 21 March 2025) were used to explore patient survival and the correlations among core targets in HCC. Survival analysis was conducted using the Kaplan–Meier method to evaluate the prognostic value of core target mRNA expression for patient survival. The significance threshold was set at *P* value < 0.05, indicating a significant association between gene expression levels and patient survival.

### Single cell RNA sequencing (scRNA-seq) analysis

The Tumor Immune Single-cell Hub 2 (TISCH2) (accessed on 22 March 2025) was used to acquire Single-cell transcriptomic datasets for the expression distribution of core target genes in the TME of HCC. Two HCC scRNA-seq datasets from TISCH2-GSE140228 (containing five samples with a total of 62, 530 cells) and GSE166635 (containing two samples with a total of 22, 631 cells)-were employed for enrichment analysis of core genes.

### Drug-likeness and toxicity profiles evaluation

The undesirable pharmacokinetic properties and toxicity of candidate compounds are among the primary factors contributing to drug development failure. The absorption, distribution, metabolism, excretion, and toxicity (ADMET) of chemical entities should be assessed as early as possible in the drug discovery process. SwissADME is a widely used web server specifically designed for predicting and analyzing the similarity, physicochemical properties, pharmacokinetic characteristics, and feasibility of small molecule synthesis (Daina et al. 2017). It is one of the most widely adopted tools in the early stages of drug design and development, enabling researchers to efficiently screen promising candidate drug molecules using Lipinski’s rule of five. ProTox 3.0 is a web server that uses machine learning models to estimate the toxicity endpoints of small molecules (Banerjee et al. 2024). It covers various toxicity endpoints, such as hepatotoxicity, neurotoxicity, nephrotoxicity, respiratory toxicity, cardiotoxicity, carcinogenicity, immunotoxicity, mutagenicity, and cytotoxicity. Thus, the drug-likeness and toxicity of key metabolites were measured according to the respective workflows of SwissADME and ProTox 3.0.

### Molecular docking analysis

The CB-DOCK2 was used for molecular docking simulations according to previous reports (Liu et al. 2022b). Firstly, the receptor proteins for the core target proteins were downloaded from the PDB database (accessed on 21 March 2025), and saved them in PDB Format. Moreover, 2-dimensional (2D) structures of ligand molecules (metabolites) were downloaded from the PubChem database(accessed on 25 March 2025), and saved them in SDF Format. Secondly, the target proteins and ligand compounds were uploaded into the CB-Dock2 (accessed on 25 March 2025) for autoblind docking analysis. Lastly, the binding ability of the metabolites to bind to the targets was confirmed and via visual analyses, in which the docking site with the lowest Vina score was selected as the optimal docking method, which provides an interactive 3D visualization of binding patterns.

CB-Dock2 was used for molecular docking simulations according to previous reports [25]. First, the core target proteins were downloaded from the PDB (accessed on 21 March 2025) and saved in PDB format. Second, the 2D structures of the metabolites were downloaded from PubChem (accessed on 25 March 2025) and saved in SDF format. Third, the target proteins and ligands were uploaded to CB-Dock2 (accessed on 25 March 2025) for auto-blind docking analysis. Finally, the binding affinity between metabolites and targets was confirmed via visual analysis. The docking site with the lowest Vina score was selected as the optimal binding pose, and CB-Dock2 provided interactive 3D visualizations of the proteins–metabolites binding patterns.

## Results

### Data collection and target mining

**Fig. 1:**
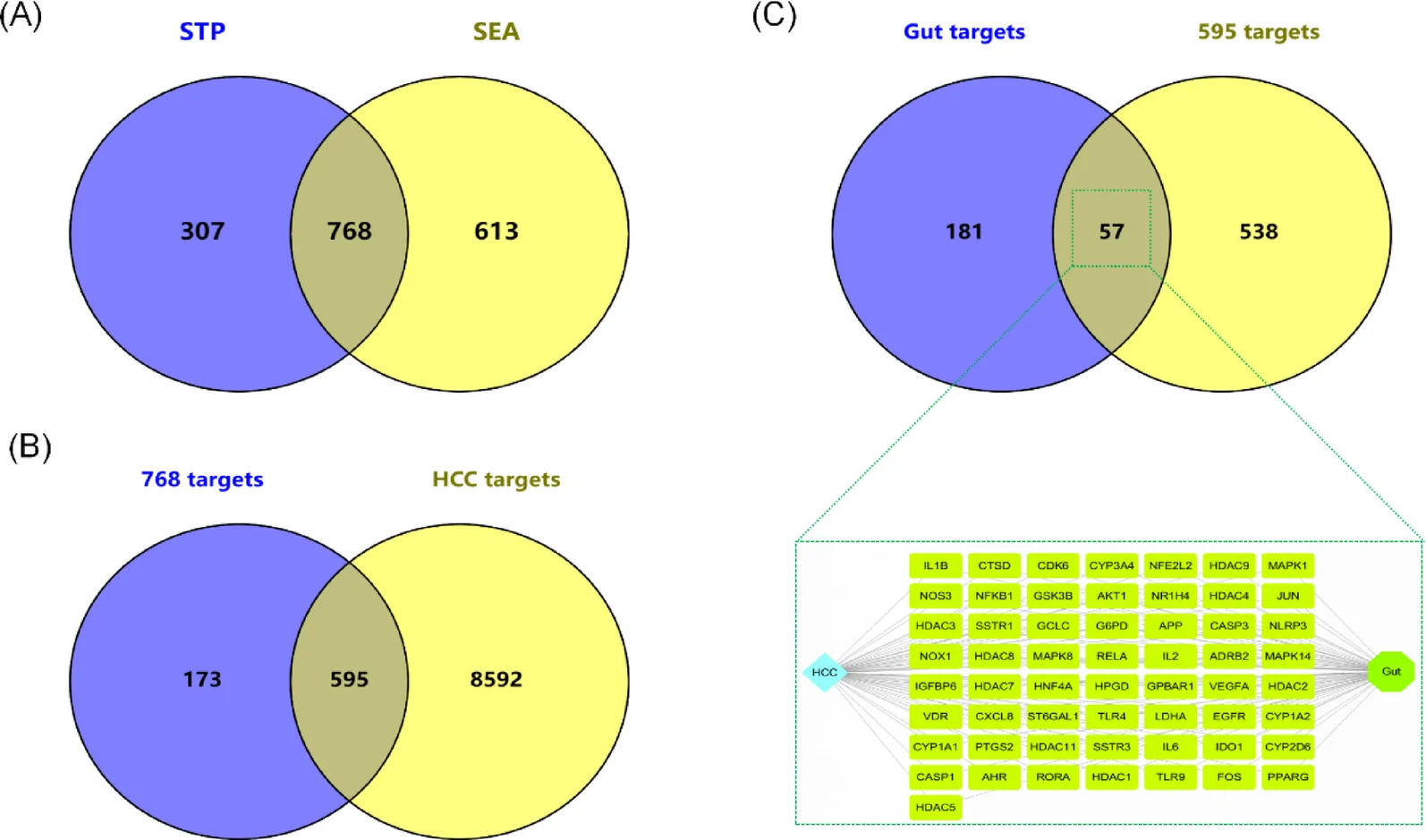
Screening and intersection study of potential targets for gut microbiota-derived metabolites and HCC-related Targets. **A** Venn diagram showing 768 common targets between the SEA and STP databases. **B** Venn diagram showing 595 common targets obtained by intersecting the SEA-STP targets with HCC-related genes. (C) Identification of 57 common targets after overlapping the 595 targets with 238 human targets of GM-derived metabolites. *Note:* SEA, Similarity Ensemble Approach; STP, Swiss Target Prediction; HCC, Hepatocellular carcinoma; “Gut targets” refer to human targets of gut microbiota-derived metabolites retrieved from the gutMGene database

Firstly, we retrieved 277 metabolites and 238 human GM targets from the gutMGene v2.0. We subsequently obtained 768 overlapping targets of GM-derived metabolites from the STP (1076 targets) and SEA (1381 targets), which were identified as potential targets of these metabolites (Fig. 1A). Moreover, we obtained a total of 9, 261 targets from the GeneCards (9, 157 targets), OMIM (36 targets), DisGeNET (30 targets) and TTD (38 targets), and 9, 187 HCC-related targets were retained after removing duplicates. After the 768 GM-derived metabolite targets were intersected with these HCC-related targets, 595 common targets were obtained (Fig. 1B). Finally, we obtained 57 common targets from the Venn diagram of 595 common targets and 238 targets in the gutMGene v2.0 (Fig. 1C).

### PPI network construction and analysis

**Fig. 2:**
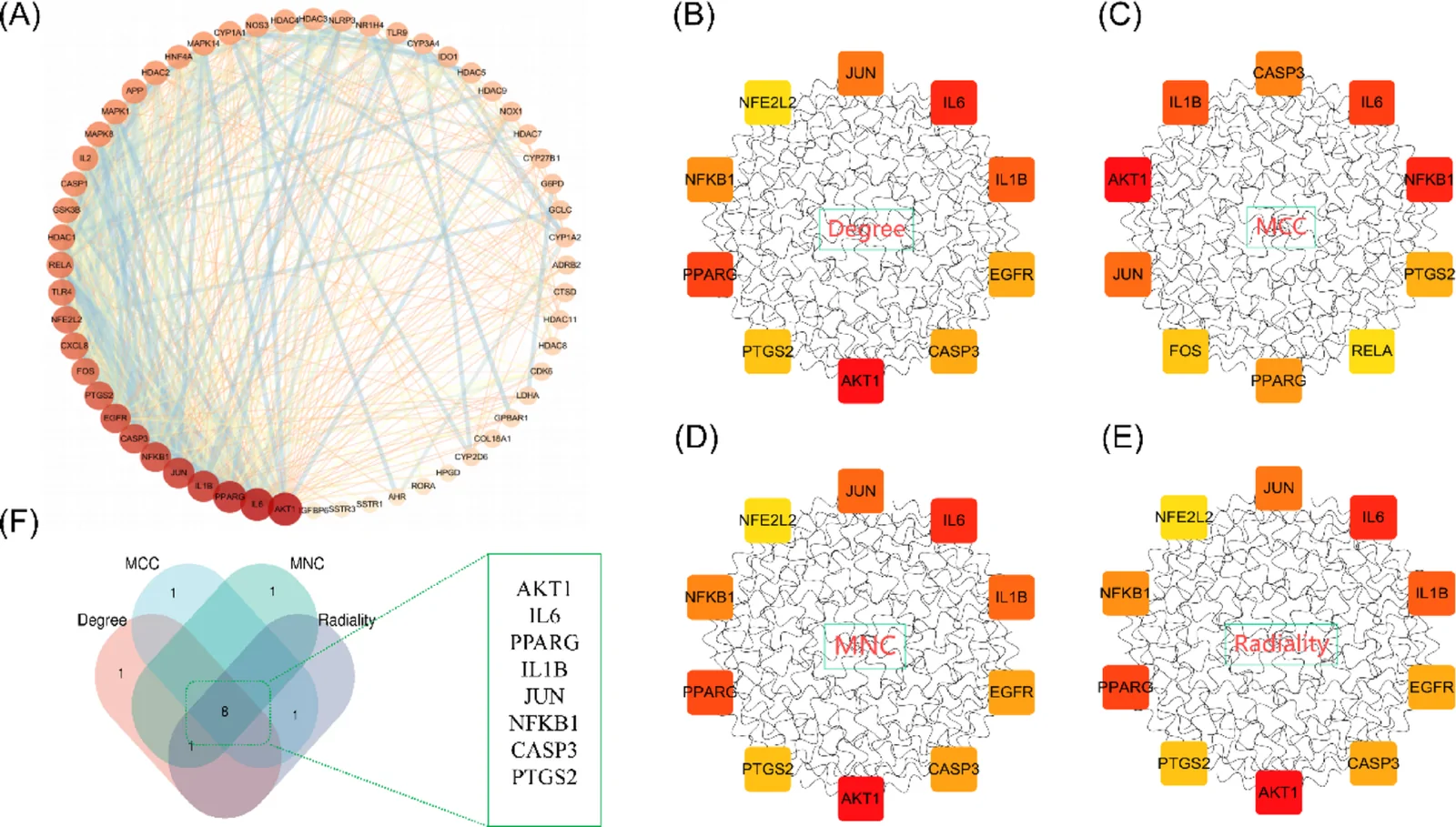
PPI network analysis. **A** PPI network visualized using Cytoscape software. **B**-**E** Top 10 hub genes identified from the 57 common targets using Degree (**B**), MCC (**C**), MNC (**D**), and Radiality (**E**) algorithms. **F** Venn diagram showing the eight overlapping hub genes identified by all four algorithms

The 57 common targets were imported into STRING to obtain the PPI network. We obtained a PPI network with 57 nodes and 514 edges, whereas a free protein (ST6GAL1) did not interact with other proteins (Fig. 2A). This network was visualized in Cytoscape, with node colors graded from dark to light according to decreasing degree values. To further identify the core targets of GM-derived metabolites involved in regulating HCC, we conducted an in-depth analysis of the network topological properties using the cytoHubba plugin. The top 10 targets were identified using the Degree, MCC, MNC, and Radiality algorithms, as illustrated in Fig. 2B–E (Gong et al. 2024). Through a comparative analysis of these algorithms, eight targets were identified: AKT1, IL6, PPARG, IL1B, JUN, NFKB1, CASP3, and PTGS2, which may be core targets of GM-derived metabolites in the treatment of HCC (Fig. 2F).

To further verify the robustness of the hub target selection, we performed a sensitivity analysis by reconstructing the PPI network using a more stringent confidence threshold of 0.7. The same four topological algorithms were applied, and the resulting core targets remained largely consistent with those identified under the 0.4 threshold, with most targets still ranked among the top candidates. Detailed results are provided in Fig. S1.

### GO functional and signaling pathway enrichment analyses

**Fig. 3:**
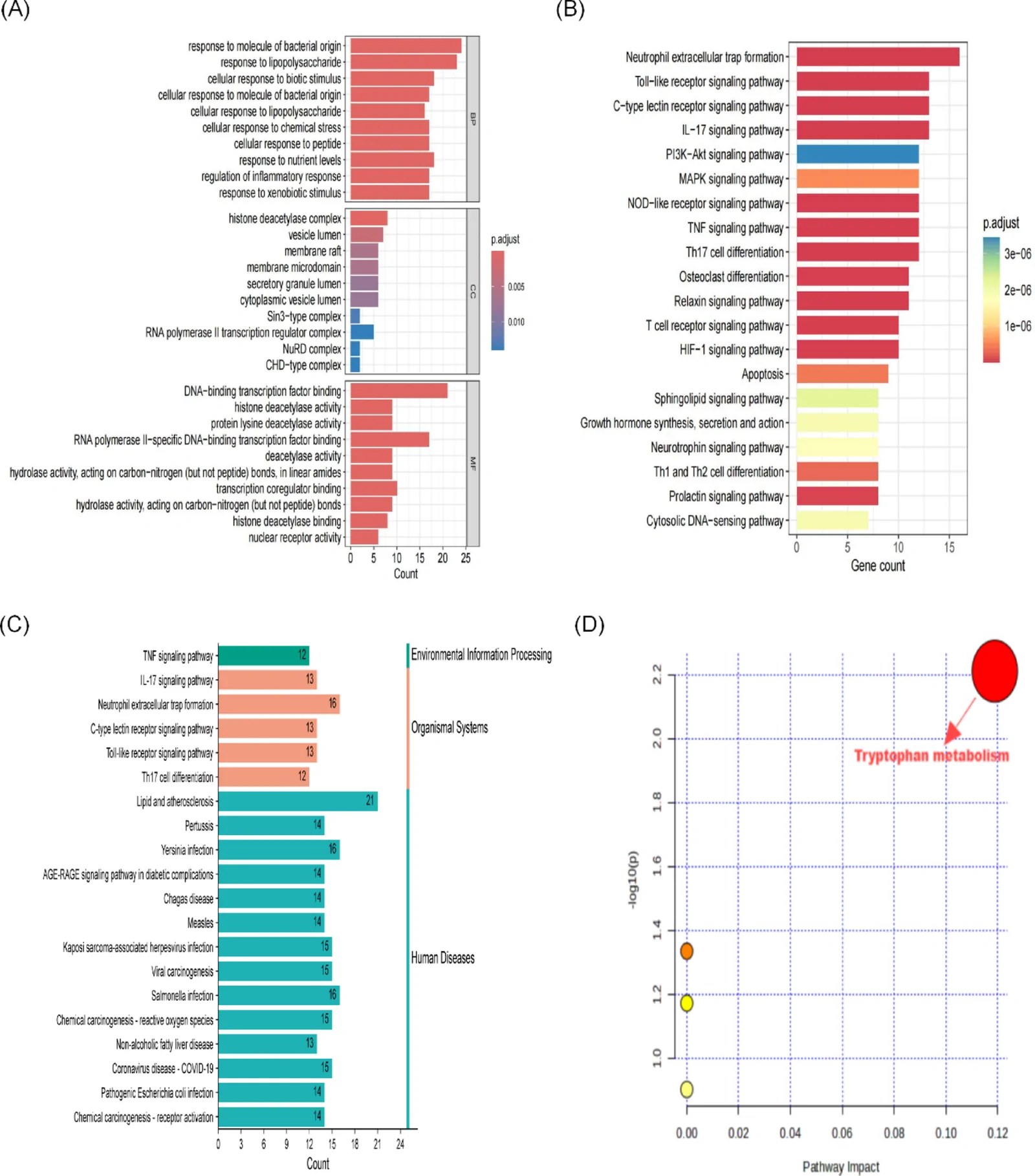
GO functional and pathway enrichment analyses. **A** Bar plot showing the enriched GO terms. **B** Bar plot of the top 20 pathways identified by KEGG enrichment analysis. **C** Bar plot of KEGG functional annotation analysis. **D** Metabolic pathway analysis of the 33 metabolites mapped to the 57 common targets. In panel D, the color gradient from yellow to red indicates decreasing p-values, while the dot size reflects the pathway impact score derived from topology analysis

GO functional and KEGG pathway enrichment analyses revealed that these hub targets regulated HCC through different pathways. We obtained a total of 1,434 GO terms and 70 KEGG pathways by selecting significantly enriched terms with *P*- values < 0.05 and *Q*-values< 0.05. The top 10 GO terms with the lowest *P*-values were selected and visualized as bar plots, as illustrated in Fig. 3A. The BP category mainly includes response to molecules of bacterial origin, response to lipopolysaccharide, and cellular response to biotic stimulus. The CC category mainly includes vesicle lumen, membrane raft, and membrane microdomain. The MF category mainly involves DNA-binding transcription factor binding, histone deacetylase activity, and protein lysine deacetylase activity. The top 20 KEGG terms with the lowest *P*-values were selected and visualized as bar plots, as illustrated in Fig. 3B and C. The enriched KEGG pathways included the Toll-like receptor signaling pathway (hsa04620), C-type lectin receptor signaling pathway (hsa04625), and IL-17 signaling pathway (hsa04657).

Furthermore, 33 metabolites corresponding to the 57 core targets were input into MetaboAnalyst 6.0 for metabolic pathway analysis, and pathway impacts > 0.1 were selected as potential metabolic pathways. We observed that only the tryptophan metabolic pathway was significantly enriched in this study (Fig. 3D). Given that the catabolic arm of this pathwayparticularly the kynurenine pathway-is well recognized for its immunosuppressive functions in the TME, these findings suggested that GM-derived metabolites may regulate HCC through tryptophan-mediated metabolic reprogramming.

### M-M-T-D network diagram construction

**Fig. 4:**
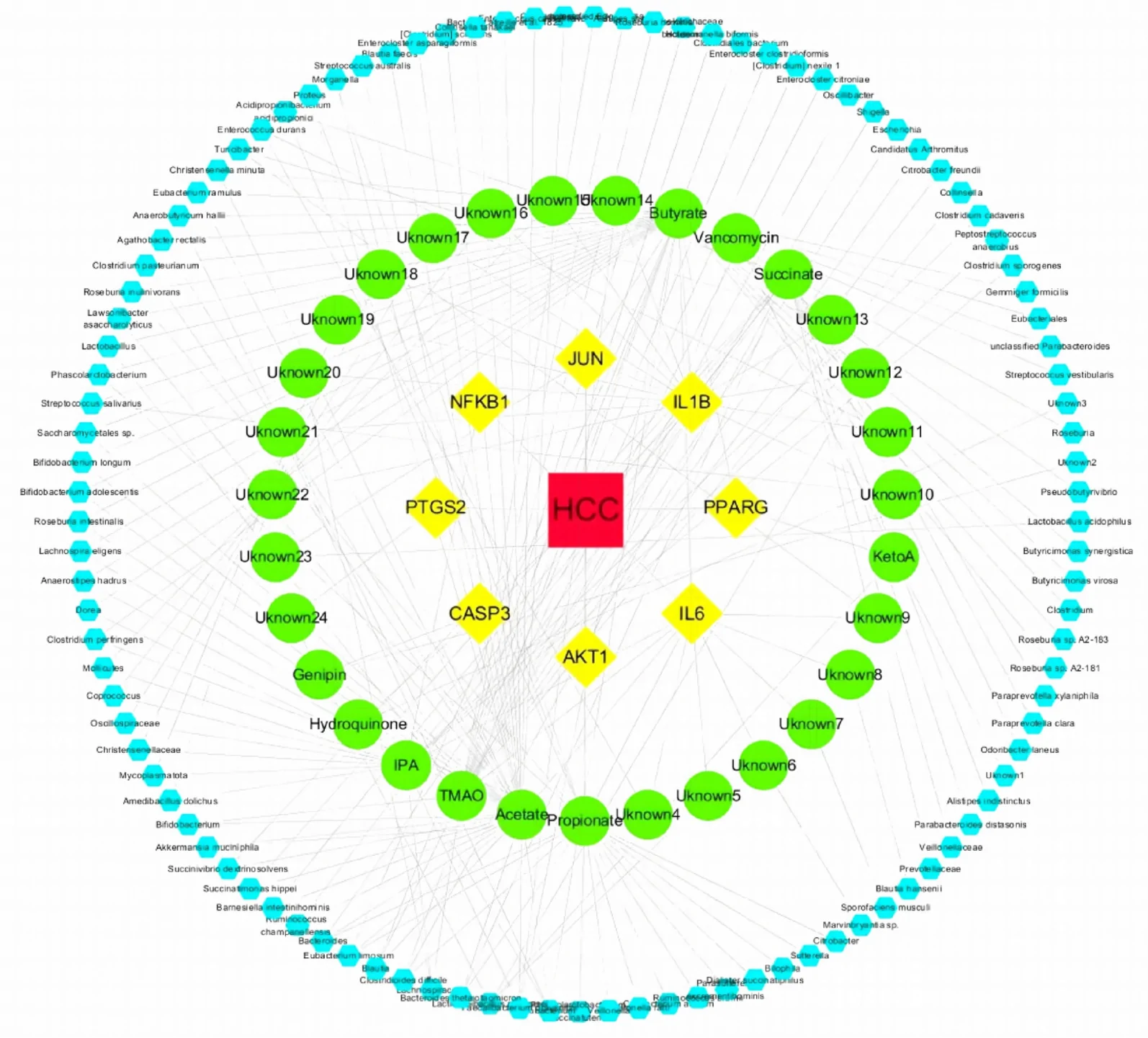
M-M-T-D network diagram. The network consists of 105 microbiota nodes (blue hexagons), 31 metabolite nodes (green ellipses), 8 target nodes (orange diamonds), and 1 disease node (red rectangle), visualizing the multi-layer regulatory relationships among these components

The M-M-T-D network diagram illustrates the relationships among microbiota (105 nodes, blue hexagon), metabolites (31 nodes, green ellipse), targets (8 nodes, orange diamond), and disease (one nodes, red rectangle) (Fig. 4).We identified 31 metabolites and 105 GM associated with the 8 core targets that regulate HCC. Furthermore, We found that the three metabolites (acetate, propionate and butyrate) had the highest degree values in the network. These SCFAs are important microbial fermentation metabolites that regulate many various aspects of human physiology (Mann et al. 2024).

### Clinical relevance analysis

**Fig. 5:**
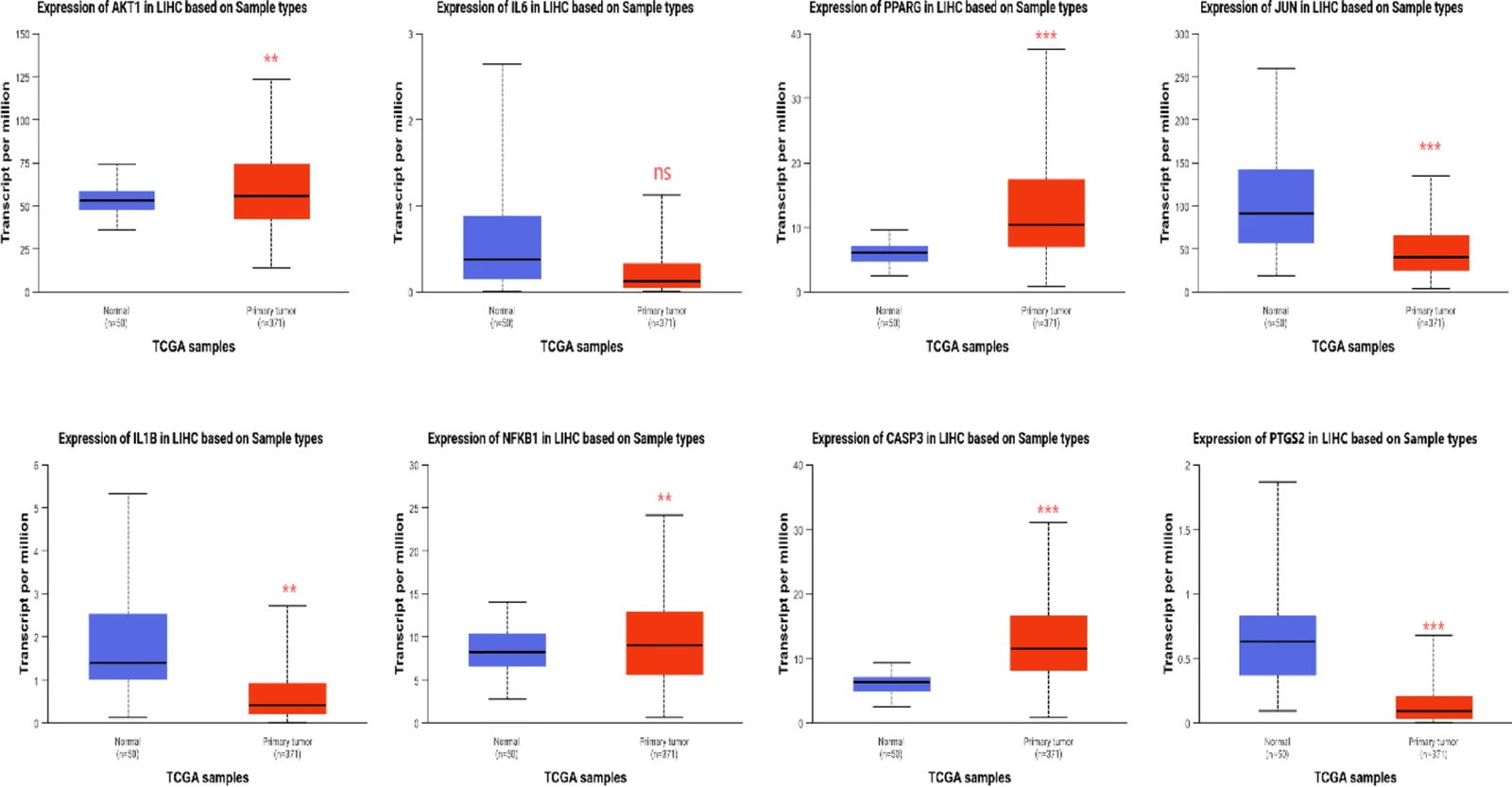
Comparison of core gene mRNA expression levels between HCC tumor tissues and normal controls using the UALCAN online server

**Fig. 6:**
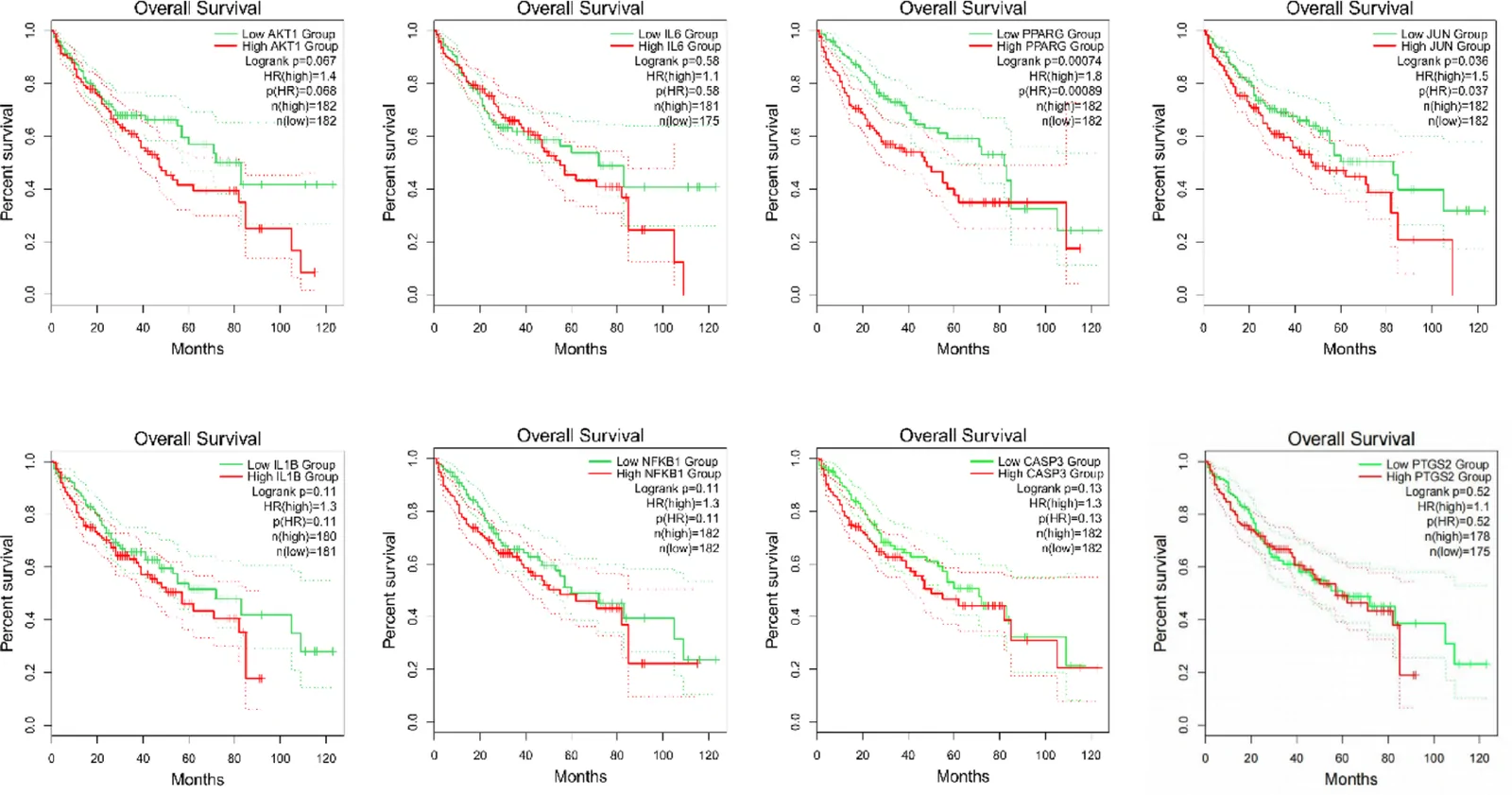
Overall survival curves showing the correlation between core gene expression levels and patient survival outcomes in HCC (GEPIA2)

The UALCAN database, which incorporates datasets from TCGA, was used to analyze the expression levels of core genes in HCC tissues and normal control samples. As illustrated in Fig. 5, the expression of these genes was significantly dysregulated in HCC tissues, indicating their potential relevance in the occurrence of liver cancer. Furthermore, survival analysis revealed that high expression of PPARG was associated with significantly shorter overall survival (log-rank *p* = 0.00074; HR = 1.8; *p* = 0.00089), while low expression of JUN was a favorable prognostic factor (log-rank *p* = 0.036; HR = 1.5; *p* = 0.037; Fig. 6). Thus, these two core targets were found to be associated with HCC patient prognosis, further highlighting their critical role in HCC pathophysiology.

### ScRNA-seq analysis

**Fig. 7:**
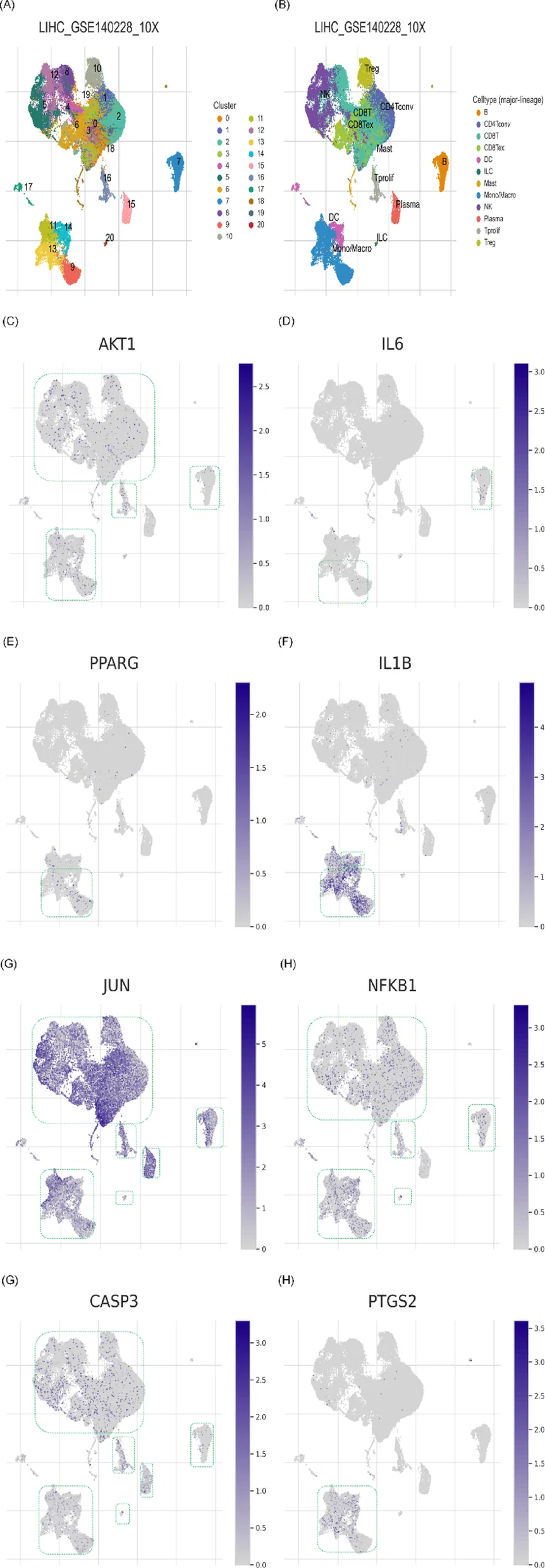
ScRNA-seq analysis of core gene expression in HCC TME (LIHC_GSE140228, TISCH2). **A** UMAP plot showing 20 unified cell clusters. **B** UMAP plot showing 12 annotated cell subsets. **C**-**H** Feature plots showing expression distribution of eight core genes across TME subsets

Given the abnormal expression levels and prognostic value of the core target genes in HCC tissues, we performed scRNA-seq data analysis to identify the specific cell types in which they are enriched. Using the HCC single-cell dataset GSE140228 from the TISCH2 database, we conducted clustering analysis, cell subgroup annotation, and gene enrichment evaluation. The results showed that the cells could be classified into 20 clusters through single-cell clustering (Fig. 7A). These clusters could further be divided into 12 cell types: B, CD4+ Tconv, CD8+ T, CD8+ Tex, DC, ILC, Mast, Mono/Macro, NK, Plasma, Tprolif, and Treg cells (Fig. 7B). The expression distribution of eight core genes across these TME subgroups is presented in Fig. 7C–H. As summarized in Table 2, AKT1, NFKB1, and CASP3 were predominantly enriched in DC, Mono/Macro cells, NK, CD4+/CD8+ T, Treg, and B cells, whereas PPARG and IL6 showed only weak expression in Mono/Macro cells. IL1B and PTGS2 were detected predominantly in DC and Mono/Macro cells. Notably, JUN was not only widely expressed in DC and Mono/Macro cells, but also significantly upregulated in NK, CD4+/CD8+ T, Treg, Mast, and B cells.

**Table 2:** Cell‑type‑specific expression distribution of core genes in HCC scRNA‑seq dataset GSE140228 (TISCH2)

Gene	TME cell cluster
AKT1	Treg, CD4/CD8 T cells, B cells, Mono/Macro, DC, NK, Tprolif, Mast
IL6	B cells, Mono/Macro
PPARG	Mono/Macro
IL1B	Treg, CD4/CD8T cells, Mono/Macro, DC, NK, Tprolif
JUN	Treg, CD4/CD8T cells, B cells, Mono/Macro, DC, NK, Tprolif, Mast, ILC, Plasma
NFKB1	Treg, CD4/CD8T cells, B cells, Mono/Macro, DC, NK, Mast, Tprolif, ILC
CASP3	Treg, CD4/CD8T cells, B cells, Mono/Macro, DC, NK, Mast, Tprolif, ILC, Plasma
PTGS2	Mono/Macro, DC

**Fig. 8:**
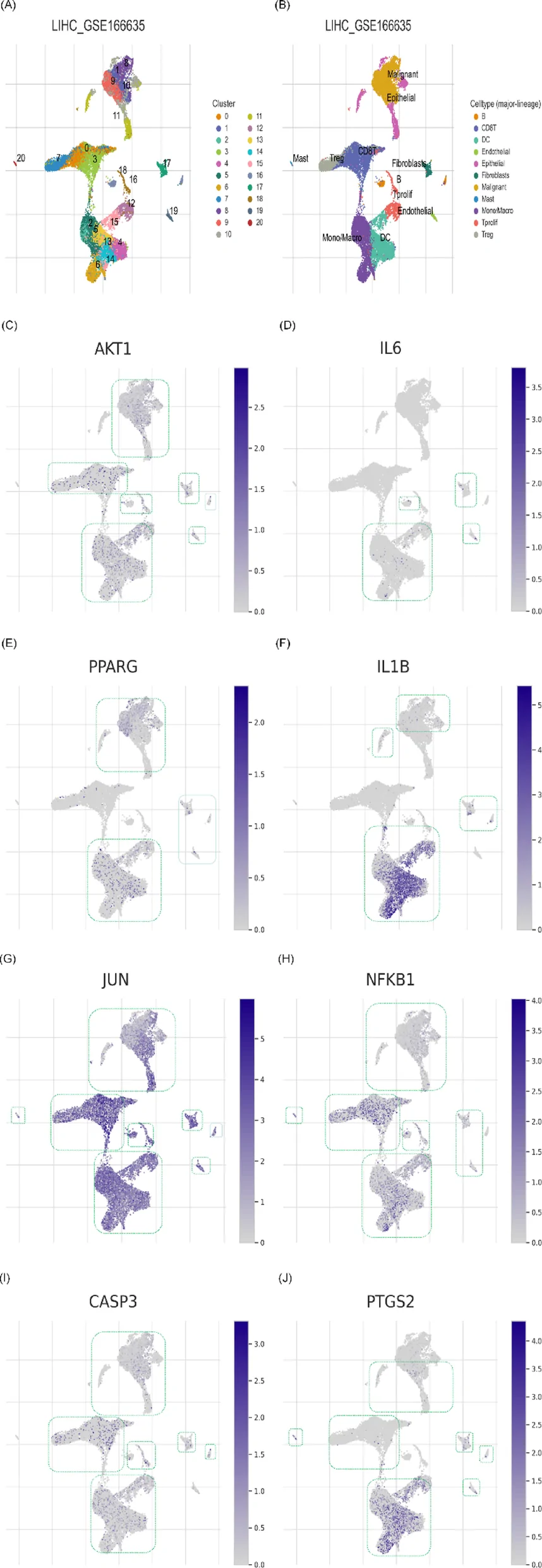
ScRNA-seq analysis of core gene expression in HCC TME (LIHC_GSE166635, TISCH2). **A** UMAP plot showing 20 unified cell clusters. **B** UMAP plot showing 11 annotated cell subsets. **C**-**H** Feature plots showing expression distribution of eight core genes across TME subsets

To verify the above findings, we analyzed a public HCC single-cell dataset (GSE166635). Analysis identified 20 cell clusters and 11 cell types in the HCC tissues (Fig. 8A, B). Figure 8C–H illustrate the expression distributions of these eight core genes across various subgroups of the TME. Consistently, AKT1, NFKB1, and CASP3 were significantly enriched in DCs, Mono/Macro cells, CD8+ T cells, and Treg cells, while PPARG, IL1B, and PTGS2 were significantly enriched in DCs and Mono/Macro cells. IL6 was expressed only at low levels in Mono/Macro cells, endothelial cells, and B cells. JUN was not only broadly detected in DCs, Mono/Macro cells, CD8+ T cells, and Treg cells, but also significantly expressed in endothelial cells, malignant cells, epithelial cells, fibroblasts, mast cells, Tprolif cells, and B cells.

### Drug-likeness and toxicity profiles evaluation

To ensure the safety of the main metabolites in therapeutic applications, we further evaluated their drug-likeness and toxicity. As summarized in Table S2, the stepwise screening from 277 initially retrieved metabolites to the final 10 candidates followed sequential filtering criteria. Back-mapping to the 8 core targets identified 31 associated metabolites, of which 21 were "Unknown" entries without defined chemical structures and were excluded, leaving 10 named metabolites (Fig. 4). Of these 10, three metabolites-TMAO, hydroquinone, and vancomycin-were further excluded due to documented toxicity, ultimately yielding 7 candidates for molecular docking and ADMET analysis. Specifically, an elevated level of circulating TMAO can induce chronic kidney disease (CKD) and cardiovascular disease (Caradonna et al. 2025). Hydroquinone, which is mainly derived from diet and GM, is a causal factor in leukemia (McDonald et al. 2001). Oral vancomycin reduces the diversity of the GM and disrupts its metabolites, such as SCFAs, and the intestinal barrier (Basolo 2020). Therefore, TMAO, hydroquinone, and vancomycin were excluded from further analysis. The chemical structures of the seven candidate metabolites are shown in Fig. S2. As shown in Table 3, IPA, KetoA, genipin, succinate, acetate, propionate, and butyrate all comply with Lipinski’s rule of five. Toxicity testing showed that IPA, KetoA, genipin, succinate, acetate, propionate, and butyrate did not cause liver damage or carcinogenicity (Table 4). These results demonstrate that these metabolites hold promise for HCC treatment.

**Table 3:** The physicochemical properties of the metabolites from gut microbiota

No	Metabolite	Lipinski Rules				Lipinski’s Violations	Bioavailability score	TPSA(Å^2^)
		MW(g/mol)	HBA	HBD	MLOGP			
		<500	<10	≤ 5	≤ 4.15	≤ 1	> 0.1	<140
1	IPA	189.21	2	2	1.4	0	0.85	53.09
2	KetoA	296.44	3	1	3.59	0	0.85	54.37
3	Genipin	226.23	5	2	0.12	0	0.56	75.99
4	Succinate	116.07	4	0	-0.54	0	0.56	80.26
5	Acetate	59.04	2	0	-0.49	0	0.55	40.13
6	Butyrate	87.10	2	0	0.49	0	0.85	40.13
7	Propionate	73.07	2	0	0.03	0	0.85	40.13

IPA, 3-Indolepropionic acid; KetoA, 10-oxo-12(Z)-octadecenoic acid; MW, molecular weight; HBA, hydrogen bond acceptor; HBD, hydrogen bond donor; MLog P, Moriguchi octanol–water partition coefficient; TPSA, topological polar surface area

**Table 4:** Toxicological properties of the metabolites from the gut microbiota

Parameter	Metabolite						
	IPA	KetoA	Genipin	Succinate	Acetate	Propionate	Butyrate
LD50 (mg/kg)	1000	11,800	237	2260	333	300	91
Hepatotoxicity	MA	MI	Inactive	Inactive	Inactive	Inactive	MI
Neurotoxicity	MI	Inactive	Inactive	Inactive	MI	Inactive	Inactive
Nephrotoxicity	MA	MI	MA	MA	MI	MI	MI
Respiratorytoxicity	MA	MI	MA	Inactive	Inactive	Inactive	Inactive
Cardiotoxicity	MI	Inactive	MI	Inactive	Inactive	Inactive	Inactive
Carcinogenicity	Inactive	MI	MI	Inactive	MI	MI	MI
Immunotoxicity	Inactive	Inactive	Inactive	Inactive	Inactive	Inactive	Inactive
Mutagenicity	Inactive	Inactive	MI	Inactive	Inactive	Inactive	Inactive
Cytotoxicity	Inactive	Inactive	Inactive	Inactive	Inactive	Inactive	Inactive

IPA, 3-Indolepropionic acid; KetoA, 10-oxo-12(Z)-octadecenoic acid; MA, Moderately Active; MI, Moderately Inactive; Inactive (Probability>= 0.7); Moderately Inactive (MI) (Probability< 0.7); Active (Probability>= 0.7); Moderately Active (MA) (Probability< 0.7)

### Molecular docking analysis

**Fig. 9:**
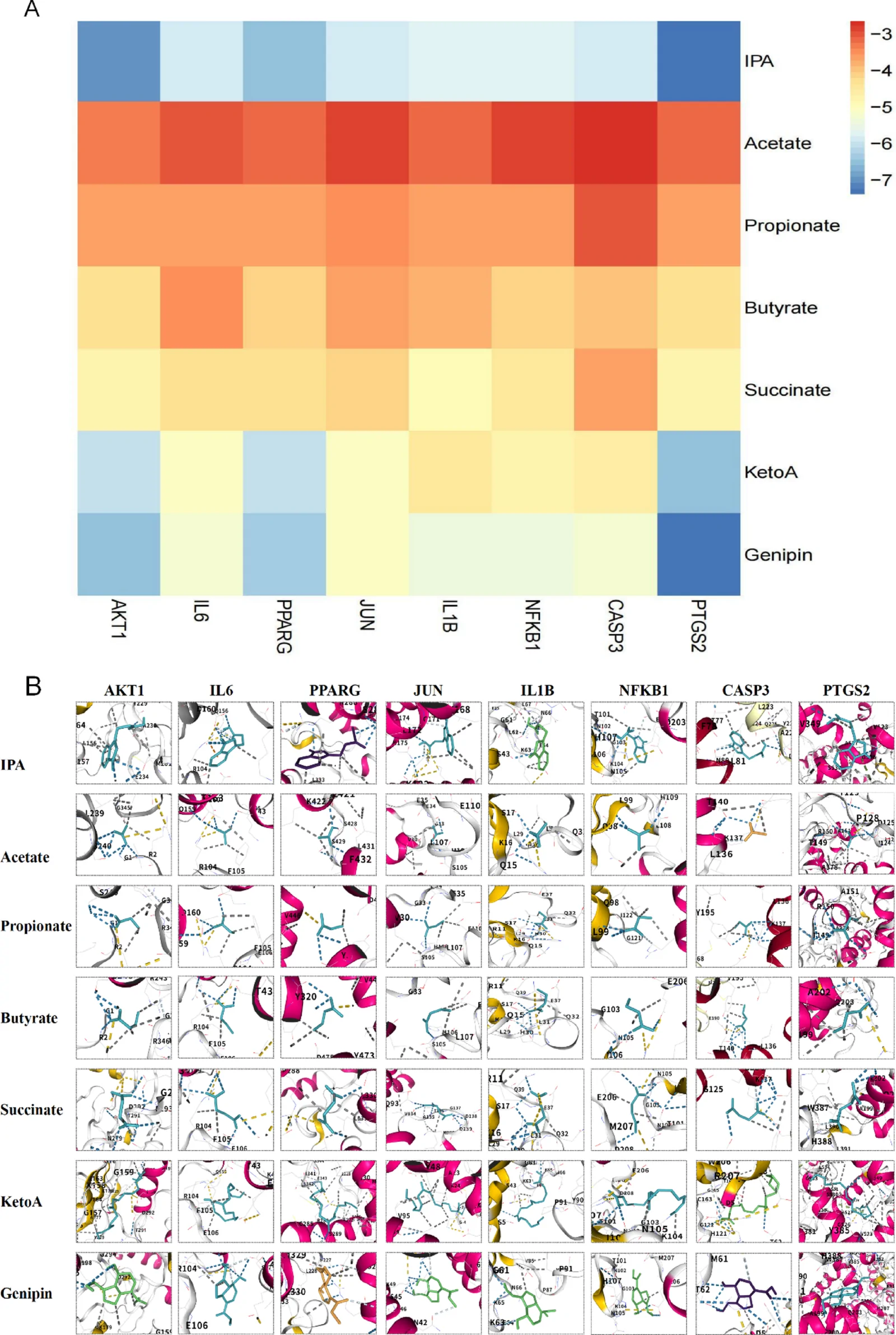
Molecular docking validation. **A** The heatmap of molecular docking binding energy. **B** Molecular docking diagram of core metabolite(s) and target(s) pairs

We evaluated the PPI network and KEGG pathway enrichment results and identified the corresponding metabolite–target pairs. These targets included AKT1, IL6, PPARG, IL1B, JUN, NFKB1, CASP3, and PTGS2, which are closely associated with HCC pathophysiology. These metabolites included IPA, KetoA, genipin, succinate, acetate, propionate, and butyrate. The binding energy quantifies the interaction strength between a ligand and a receptor (Pinzi and Rastelli 2019). Notably, a lower binding affinity indicates a stronger binding interaction. The heatmap displays the affinity between the metabolites and targets (Fig. 9A). The docking results show that the genipin–PTGS2 pair exhibits the strongest binding affinity (− 7.4 kcal/mol), followed by the IPA–AKT1 pair (− 7.0 kcal/mol). Figure 9B illustrates the formation of hydrogen bonds between the metabolites and specific active-site residues of the target proteins. We summarize the analysis results in Tables S3 and S4, which show that these metabolites have potential biological activities in the regulation of HCC, especially IPA, KetoA, and genipin.

## Discussion

In recent years, studies on the gut-liver axis have provided new insights into the pathogenesis of liver diseases, such as NAFLD, ALD, and HCC (Tilg et al. 2022). The gut–liver axis represents the bidirectional communication between the GM, the intestinal barrier, and the liver. This reciprocal interaction is established via the portal vein, which transports GM-derived products directly to the liver, and via the biliary and antibody secretion routes, which provide feedback from the liver to the intestinal barrier (Tripathi et al. 2018; Wang et al. 2021). The role of GM-derived metabolites in tumor therapy has attracted considerable attention. Bile acids (BAs) have been identified as key signaling molecules that regulate liver disease progression, and secondary BAs such as deoxycholic acid (DCA) can induce chronic inflammation by activating inflammatory pathways such as NF-κB, thus promoting the development of liver cancer (Albillos et al. 2020; Wang et al. 2025a). Conversely, beneficial metabolites represented by butyrate can not only effectively reduce inflammatory responses, but also enhance the integrity of the intestinal barrier, thereby blocking the translocation of pro-tumor metabolites and reducing the risk of HCC (Lv et al. 2025).

In an to explore the potential beneficial effects of gut microbial metabolites in the treatment of HCC, this investigation utilized a multi-faceted strategy that included network pharmacology, molecular docking, and single-cell sequencing analysis. We confirmed that KetoA, IPA, genipin, succinate and SCFAs (including: acetate, propionate and butyrate) are promising potential metabolites with the ability to regulate HCC. KetoA is produced by the intestinal metabolism of *Lacticaseibacillus paracase*i. *Lactobacillus paracasei sub* sp.* paracasei* M5L (M5-EPSs) is an important property in the field of antitumor research that can enhance apoptosis and inhibit the proliferation of tumor cells by regulating the Bcl-2 family proteins, producing reactive oxygen species (ROS) and cell wall proteins, enhancing calreticulin translocation, and modifying the cell cycle (Hu et al. 2015). IPA is a specific metabolite that is produced mainly by the *Clostridium* spp., *Bacteroides* spp., *Lactobacillus* spp.*,*etc. Preclinical studies have shown that IPA has anti-inflammatory properties, protects the integrity of the intestinal barrier, and inhibits cell proliferation of NASH–HCC cell lines (Gao et al. 2025; Zhang et al. 2021). In patient-derived organoid models, IPA (5 mM) improved the responsiveness to anti-PD-1 treatment at the pan-cancer level, including melanoma, breast cancer and colorectal cancer (Jia et al. 2024). The therapeutic effect of genipin in a patient-derived HCC xenograft nude mouse model has been confirmed, which may be attributed to the interaction of this compound with the Src homology-2 (SH2) domain, which suppresses the activity of signal transducer and activator of transcription-3 (STAT-3), thereby blocking the abnormal activation of related signaling pathways (Hong et al. 2020). In addition, we confirmed that these metabolites are promising postbiotics on the based of drug‒likeness, toxicity and molecular docking evaluations. SCFAs are metabolites produced from the beneficial bacterial fermentation of dietary fibers in the gastrointestinal microbiome and include mainly acetate, propionate and butyrate, etc. (Mann et al. 2024). Recent studies have demonstrated that SCFAs exert anticancer effects by promoting apoptosis and cell cycle arrest in cancer cells, thereby preventing their metastasis and invasion (Li et al. 2025). Previous studies have demonstrated that butyrate levels are lower in the plasma of patients with HCC than in that of healthy individuals. Butyrate supplementation or depletion of the short-chain acyl-CoA dehydrogenase (SCAD) gene (ACADS, encoding a key enzyme for butyrate metabolism) could significantly inhibits HCC proliferation and metastasis,and butyrate supplementation could improve the therapeutic efficacy of the tyrosine kinase inhibitor sorafenib (Che et al. 2023)*. Lactobacillus reuteri* was markedly reduced in HCC xenograft mouse model, accompanied by decreased SCFAs levels(especially acetate), whereas transplantation of *L. reuteri* increases acetate levels, decreases IL-17A secretion, and inhibits the growth of HCC (Hu et al. 2023). Propionate may improve HCC not only through a cAMP level dependent pathway, but also through binding to GPR43 (Bindels et al. 2012)**.**

Through PPI network analysis, we ultimately identified that AKT1, IL6, PPARG, JUN, IL1B, NFKB1, CASP3 and PTGS2 as core targets that may be involved in the mechanism of HCC. We validated the differential expression of the core targets and their prognostic value for HCC patient survival using online databases, verified their cell-specific expression patterns, and further confirmed their direct interactions with metabolites via molecular docking analysis. Based on online resources analysis, we found that these core genes are closely associated with the development and progression of HCC. In this study, we found that high PPARG expression was significantly associated with clinical outcomes in HCC patients. According to previous reports, PPARγ is a potential therapeutic target for HCC, and selective blockade of PPARγ can inhibit metabolic pathways that promote hepatic tumorigenesis (Patitucci et al. 2017). However, some studies have demonstrated that high PPARG expression can inhibit the Wnt/β-catenin pathway, induce tumor cell apoptosis, or suppress the inflammatory microenvironment to delay HCC progression (To et al. 2021). Different etiologies and molecular subtypes may affect the functional role of PPARG. We further focused on the roles of these genes in HCC and the effects of gut microbial metabolites on them. Using the UALCAN database with TCGA-LIHC cohort data, we found that the mRNA expression level of JUN was significantly higher in normal liver tissues than in primary HCC tissues. Further Kaplan–Meier survival analysis revealed that HCC patients with high intratumoral JUN expression exhibited markedly shorter overall survival than those with low JUN expression. These findings reflect a context-dependent dual role of JUN. In normal liver, JUN primarily maintains hepatic homeostasis and exerts tumor-suppressive effects (Kurlishchuk et al. 2024). Following HCC initiation, however, elevated JUN expression drives oncogenic signaling and serves as a risk factor for poor prognosis in HCC patients (Eferl et al. 2003).

GO enrichment analysis indicated that these GM metabolites might play pivotal roles in the regulation of oxidative stress, inflammation and cell proliferation. Anti-inflammatory and antioxidant effects are the core strategies for preventing HCC. Chronic inflammation triggers excessive ROS production by recruiting immune cells (such as macrophages), and oxidative stress can enhance inflammatory signals, forming a vicious cycle and accelerating HCC progression (Gaul et al. 2021; Zhan et al. 2023). KEGG pathway analysis revealed that GM-derived metabolites can modulate several critical signaling pathways, such as the IL-17, C-type lectin receptors (CLRs), Toll-like receptors (TLRs), and TNF signaling pathways, suggesting their potential role in systemic immune and metabolic regulation. The expression of IL-17RA is upregulated in patients with HBV/HCV infections, nonalcoholic steatohepatitis (NASH), alcohol-associated liver disease (AALD), HCC, and experimental models of chronic toxic liver injury (Li et al. 2021b). A previous report revealed that IL-17A is a tumor-promoting cytokine that critically regulates alcohol-induced hepatic steatosis, inflammation, fibrosis, and HCC (Ma et al. 2020). Recent research has suggested that CLRs are downregulated in HCC and that their expression is correlated with the clinical prognosis of HCC patients (Xia et al. 2023). According to a previous report, activation of the TLR-4-mediated NF-κB signaling pathway can induce NLRP3 inflammasome complex formation and multiple inflammatory factor secretion in the liver, thus promoting HCC progression (Liu et al. 2022a). TNF signaling pathways are abnormally activated during liver injury and play a key role in the subsequent development of HCC (Baffy et al. 2012). Metabolic pathway analysis revealed that the tryptophan metabolic pathway was significantly enriched. Tryptophan (Trp) is an essential amino acid, and the liver is the main site of Trp metabolism, which is associated with tumor immunity. Dysregulated Trp metabolism can create an immunosuppressive microenvironment, promoting the occurrence and development of HCC (Agus et al. 2018). Trp metabolites, such as kynurenine, kynurenic acid, cinnabarinic acid, IPA, and indole−3-aldehyde, improve the integrity of the intestinal epithelium through the aryl hydrocarbon receptor (AhR) and pregnane X receptor (PXR) signaling pathways, thereby reducing LPS translocation, liver injury, and inflammation (Chen et al. 2022; Scott et al. 2020).

Although our study preliminarily elucidated the key microbiota, crucial metabolites and significant targets for HCC intervention, we analyzed only the uppermost components through the M-M-T-D network to represent crosstalk between the host and microbiota. Among the candidate metabolites, IPA, ketoA, and genipin showed strong binding affinities to their respective targets (NFKB1, IL1B, PPARG, and CASP3). While only PPARG and JUN were significantly associated with patient survival in bulk transcriptomic analysis, our single-cell data revealed that NFKB1, IL1B, and CASP3 are preferentially expressed in key immune cell populations within the HCC microenvironment, suggesting that these metabolites may exert immunomodulatory effects rather than direct tumoricidal activity. This observation highlights the value of integrating single-cell resolution into network pharmacology workflows to uncover cell-type-specific mechanisms that may be masked in bulk tissue analyses.

However, several limitations of this computational biology approach should be acknowledged. First, heterogeneity across different patient populations is not adequately captured. Second, the roles of some core genes in liver cancer were validated solely through public databases and require experimental corroboration. Third, the metabolite profiles retrieved from gutMGene v2.0 may be incomplete, and the appropriate in vivo dosage for each candidate metabolite remains to be determined. Fourth, although molecular docking provided preliminary binding affinity estimates, the lack of molecular dynamics (MD) simulations means that protein flexibility, solvent effects, and conformational entropy changes-particularly critical for highly flexible short-chain fatty acids (acetate, propionate, and butyrate)-were not evaluated. Therefore, the inferred "binding stability" for SCFAs should be interpreted with caution. Future studies incorporating explicit-solvent MD simulations and free energy perturbation calculations are warranted to more reliably assess the thermodynamic and kinetic profiles of these flexible metabolites, thereby providing a stronger foundation for candidate prioritization and experimental validation. Additionally, the proposed immunomodulatory roles of IPA, ketoA, and genipin require functional validation in appropriate in vitro and in vivo models before any translational conclusions can be drawn.

## Conclusion

In this study, we employed an integrated approach combining network pharmacology and bioinformatics to identify GM-derived metabolites with therapeutic potential for HCC. The results revealed that multiple gut microbes may serve as crucial sources of bioactive metabolites with anti-HCC effects. Through comprehensive screening and validation, we identified seven metabolites (postbiotics) with anti-HCC potential. These metabolites demonstrated multi-target synergistic regulatory effects on HCC-related pathways via interactions with host receptors and signaling molecules. Our study may open a new avenue for developing safe and effective HCC therapies. The identified candidate metabolites require further validation to assess their translational potential as therapeutic agents or as complementary interventions in HCC treatment.

## Supplementary Information

Below is the link to the electronic supplementary material.

Supplementary Material 1

Supplementary Material 2

## Data Availability

All data generated or analyzed during this study are included in this published article and its supplementary information flies.

## Abbreviations

HCC: Hepatocellular Carcinoma
PLC: Primary liver cancer
AILD: Autoimmune liver disease
ALD: Alcoholic liver disease
NAFLD: Nonalcoholic fatty liver disease
NASH: Nonalcoholic steatohepatitis
AALD: Alcohol-associated liver disease
GM: Gut Microbiota
SMILE: Simplified Molecular Input Line Entry System
OMIM: Online Mendelian Inheritance in Man
TTD: Therapeutic Target Database
PPI: Protein–protein interaction
AKT1: AKT serine/threonine kinase 1 gene
IL6: Lnterleukin-6
PPARG: Peroxisome proliferator-activated receptor gamma
JUN: Jun proto-Oncogene
IL1B: Interleukin 1 Beta
NFKB1: Nuclear factor kappa-B subunit 1
CASP3: Cysteinyl Aspartate-Specific Proteinase
EGFR: Epidermal Growth Factor Receptor
GO: Gene ontology
BP: Biological process
CC: Cellular component
MF: Molecular function
KEGG: Kyoto Encyclopedia of Genes and Genomes
TCGA: The Cancer Genome Atlas
GTEx: Genotype-Tissue Expression
SCFAs: Short-chain fatty acids
LPS: Lipopolysaccharide
VEGF: Vascular endothelial growth factor
MMTD: Microbiota-Metabolites-Targets-Disease
LD50: Lethal dose 50
HBV: Hepatitis B virus
HCV: Hepatitis C virus
TPSA: Topological Polar surface Area
GEPIA2: Gene Expression Profiling Interactive Analysis V2
IPA: 3-Indolepropionic acid
TMAO: Trimethylamine oxide
KetoA: 10-Oxo-12(Z)-octadecenoic acid
TME: Tumor microenvironment

## References

[CR1] Agus A, Planchais J, Sokol H (2018) Gut microbiota regulation of tryptophan metabolism in health and disease. Cell Host Microbe 23(6):716–724 29902437 10.1016/j.chom.2018.05.003

[CR2] Albillos A, de Gottardi A, Rescigno M (2020) The gut-liver axis in liver disease: pathophysiological basis for therapy. J Hepatol 72(3):558–577 31622696 10.1016/j.jhep.2019.10.003

[CR3] Baffy G, Brunt EM, Caldwell SH (2012) Hepatocellular carcinoma in non-alcoholic fatty liver disease: an emerging menace. J Hepatol 56(6):1384–1391 22326465 10.1016/j.jhep.2011.10.027

[CR4] Banerjee P, Kemmler E, Dunkel M, Preissner R (2024) ProTox 3.0: a webserver for the prediction of toxicity of chemicals. Nucleic Acids Res 52:W513–W520 38647086 10.1093/nar/gkae303 PMC11223834

[CR5] Basolo, A., Hohenadel, M., Ang, Q. Y., Piaggi, P., Heinitz, S., Walter, M., Walter, P., Parrington, S., Trinidad, D. D., von Schwartzenberg, R. J., Turnbaugh, P. J. & Krakoff, J. (2020) 10.1038/s41591-020-0801-z 32235930

[CR6] Bindels LB, Porporato P, Dewulf EM, Verrax J, Neyrinck AM, Martin JC, Scott KP, Buc Calderon P, Feron O, Muccioli GG, Sonveaux P, Cani PD, Delzenne NM (2012) Gut microbiota-derived propionate reduces cancer cell proliferation in the liver. Br J Cancer 107(8):1337–1344 22976799 10.1038/bjc.2012.409 PMC3494429

[CR7] Caradonna E, Abate F, Schiano E, Paparella F, Ferrara F, Vanoli E, Difruscolo R, Goffredo VM, Amato B, Setacci C, Setacci F, Novellino E (2025) Trimethylamine-N-Oxide (TMAO) as a rising-star metabolite: implications for human health. Metabolites 15(4):220 40278349 10.3390/metabo15040220 PMC12029716

[CR8] Chandrashekar DS, Karthikeyan SK, Korla PK, Patel H, Shovon AR, Athar M, Netto GJ, Qin ZS, Kumar S, Manne U, Creighton CJ, Varambally S (2022) UALCAN: an update to the integrated cancer data analysis platform. Neoplasia 25:18–27 35078134 10.1016/j.neo.2022.01.001 PMC8788199

[CR9] Che Y, Chen G, Guo Q, Duan Y, Feng H, Xia Q (2023) Gut microbial metabolite butyrate improves anticancer therapy by regulating intracellular calcium homeostasis. Hepatology 78(1):88–102 36947402 10.1097/HEP.0000000000000047

[CR10] Chen W, Wen L, Bao Y, Tang Z, Zhao J, Zhang X, Wei T, Zhang J, Ma T, Zhang Q, Zhi X, Li J, Zhang C, Ni L, Li M, Liang T (2022) Gut flora disequilibrium promotes the initiation of liver cancer by modulating tryptophan metabolism and up-regulating SREBP2. Proc Natl Acad Sci U S A 119(52):e2203894119 36534812 10.1073/pnas.2203894119 PMC9907126

[CR11] Chidambaranathan-Reghupaty S, Fisher PB, Sarkar D (2021) Hepatocellular carcinoma (HCC): epidemiology, etiology and molecular classification. Adv Cancer Res 149:1–61 33579421 10.1016/bs.acr.2020.10.001 PMC8796122

[CR12] Daina A, Michielin O, Zoete V (2017) SwissADME: a free web tool to evaluate pharmacokinetics, drug-likeness and medicinal chemistry friendliness of small molecules. Sci Rep 7:42717 28256516 10.1038/srep42717 PMC5335600

[CR13] Eferl R, Ricci R, Kenner L, Zenz R, David JP, Rath M, Wagner EF (2003) Liver tumor development. c-Jun antagonizes the proapoptotic activity of p53. Cell 112(2):181–192 12553907 10.1016/s0092-8674(03)00042-4

[CR14] Gao H, Sun M, Li A, Gu Q, Kang D, Feng Z, Li X, Wang X, Chen L, Yang H, Cong Y, Liu Z (2025) Microbiota-derived IPA alleviates intestinal mucosal inflammation through upregulating Th1/Th17 cell apoptosis in inflammatory bowel disease. Gut Microbes 17(1):2467235 39956891 10.1080/19490976.2025.2467235 PMC11834480

[CR15] Gaul S, Leszczynska A, Alegre F, Kaufmann B, Johnson CD, Adams LA, Wree A, Damm G, Seehofer D, Calvente CJ, Povero D, Kisseleva T, Eguchi A, McGeough MD, Hoffman HM, Pelegrin P, Laufs U, Feldstein AE (2021) Hepatocyte pyroptosis and release of inflammasome particles induce stellate cell activation and liver fibrosis. J Hepatol 74(1):156–167 32763266 10.1016/j.jhep.2020.07.041 PMC7749849

[CR16] Gong W, Sun P, Li X, Wang X, Zhang X, Cui H, Yang J (2024) Investigating the molecular mechanisms of resveratrol in treating cardiometabolic multimorbidity: a network pharmacology and bioinformatics approach with molecular docking validation. Nutrients 16(15):2488 39125368 10.3390/nu16152488 PMC11314475

[CR17] Hong M, Lee S, Clayton J, Yake W, Li J (2020) Genipin suppression of growth and metastasis in hepatocellular carcinoma through blocking activation of STAT-3. J Exp Clin Cancer Res 39(1):146 32741371 10.1186/s13046-020-01654-3 PMC7397684

[CR18] Hopkins AL (2007) Network pharmacology. Nat Biotechnol 25(10):1110–1111 17921993 10.1038/nbt1007-1110

[CR19] Hsu CL, Schnabl B (2023) The gut-liver axis and gut microbiota in health and liver disease. Nat Rev Microbiol 21(11):719–733 37316582 10.1038/s41579-023-00904-3 PMC10794111

[CR20] Hu P, Song W, Shan Y, Du M, Huang M, Song C, Zhang L (2015) *Lactobacillus paracasei* subsp. *paracasei* M5L induces cell cycle arrest and calreticulin translocation via the generation of reactive oxygen species in HT-29 cell apoptosis. Food Funct 6(7):2257–2265 26068306 10.1039/c5fo00248f

[CR21] Hu C, Xu B, Wang X, Wan WH, Lu J, Kong D, Jin Y, You W, Sun H, Mu X, Feng D, Chen Y (2023) Gut microbiota-derived short-chain fatty acids regulate group 3 innate lymphoid cells in HCC. Hepatology (Baltimore, MD) 77(1):48–64 35262957 10.1002/hep.32449 PMC9970019

[CR22] Jia D, Wang Q, Qi Y, Jiang Y, He J, Lin Y, Sun Y, Xu J, Chen W, Fan L, Yan R, Zhang W, Ren G, Xu C, Ge Q, Wang L, Liu W, Xu F, Wu P, Wang Y, Chen S, Wang L (2024) Microbial metabolite enhances immunotherapy efficacy by modulating T cell stemness in pan-cancer. Cell 187(7):1651-1665 e1621 38490195 10.1016/j.cell.2024.02.022

[CR23] Kang Y, Cai Y, Yang Y (2022) The gut microbiome and hepatocellular carcinoma: implications for early diagnostic biomarkers and novel therapies. Liver Cancer (Basel) 11(2):113–125 10.1159/000521358 PMC9109080 35634424

[CR24] Kurlishchuk Y, Cindric Vranesic A, Jessen M, Kipping A, Ritter C, Kim K, Cramer P, von Eyss B (2024) A non-canonical repressor function of JUN restrains YAP activity and liver cancer growth. EMBO J 43(20):4578–4603 39210147 10.1038/s44318-024-00188-0 PMC11480203

[CR25] Li C, Tang Z, Zhang W, Ye Z, Liu F (2021a) GEPIA2021: integrating multiple deconvolution-based analysis into GEPIA. Nucleic Acids Res 49(W1):W242–W246 34050758 10.1093/nar/gkab418 PMC8262695

[CR26] Li N, Yamamoto G, Fuji H, Kisseleva T (2021b) Interleukin-17 in liver disease pathogenesis. Semin Liver Dis 41(4):507–515 34130335 10.1055/s-0041-1730926

[CR27] Li S, Duan Y, Luo S, Zhou F, Wu Q, Lu Z (2025) Short-chain fatty acids and cancer. Trends Cancer 11(2):154–168 39638744 10.1016/j.trecan.2024.11.003

[CR28] Liu B, Zhou Z, Jin Y, Lu J, Feng D, Peng R, Sun H, Mu X, Li C, Chen Y (2022a) Hepatic stellate cell activation and senescence induced by intrahepatic microbiota disturbances drive progression of liver cirrhosis toward hepatocellular carcinoma. J Immunother Cancer. 10.1136/jitc-2021-003069 34996812 PMC8744134

[CR29] Liu Y, Yang X, Gan J, Chen S, Xiao ZX, Cao Y (2022b) CB-Dock2: improved protein-ligand blind docking by integrating cavity detection, docking and homologous template fitting. Nucleic Acids Res 50(W1):W159–W164 35609983 10.1093/nar/gkac394 PMC9252749

[CR30] Lv Y, Jiang M, Ouyang Y, Zheng X, Zhang L, Yao J, Hu L, Zhao J, Li Z, Wang S (2025) Sodium butyrate-loaded microspheres with enhanced bioavailability for targeted treatment of intestinal barrier injury. Adv Healthc Mater 14(9):e2402773 39629540 10.1002/adhm.202402773

[CR31] Ma HY, Yamamoto G, Xu J, Liu X, Karin D, Kim JY, Alexandrov LB, Koyama Y, Nishio T, Benner C, Heinz S, Rosenthal SB, Liang S, Sun M, Karin G, Zhao P, Brodt P, McKillop IH, Quehenberger O, Dennis E, Saltiel A, Tsukamoto H, Gao B, Karin M, Brenner DA, Kisseleva T (2020) IL-17 signaling in steatotic hepatocytes and macrophages promotes hepatocellular carcinoma in alcohol-related liver disease. J Hepatol 72(5):946–959 31899206 10.1016/j.jhep.2019.12.016 PMC7167339

[CR32] Ma H, Yang L, Liang Y, Liu F, Hu J, Zhang R, Li Y, Yuan L, Feng F (2024) *B. **thetaiotaomicron*-derived acetic acid modulate immune microenvironment and tumor growth in hepatocellular carcinoma. Gut Microbes 16(1):2297846 38270111 10.1080/19490976.2023.2297846 PMC10813637

[CR33] Mann ER, Lam YK, Uhlig HH (2024) Short-chain fatty acids: linking diet, the microbiome and immunity. Nat Rev Immunol 24(8):577–595 38565643 10.1038/s41577-024-01014-8

[CR34] McDonald TA, Holland NT, Skibola C, Duramad P, Smith MT (2001) Hypothesis: phenol and hydroquinone derived mainly from diet and gastrointestinal flora activity are causal factors in leukemia. Leukemia 15(1):10–20 11243376 10.1038/sj.leu.2401981

[CR35] Nie Q, Luo X, Wang K, Ding Y, Jia S, Zhao Q, Li M, Zhang J, Zhuo Y, Lin J, Guo C, Zhang Z, Liu H, Zeng G, You J, Sun L, Lu H, Ma M, Jia Y, Zheng MH, Pang Y, Qiao J, Jiang C (2024) Gut symbionts alleviate MASH through a secondary bile acid biosynthetic pathway. Cell 187(11):2717-2734 e2733 38653239 10.1016/j.cell.2024.03.034

[CR36] Nogales C, Mamdouh ZM, List M, Kiel C, Casas AI, Schmidt H (2022) Network pharmacology: curing causal mechanisms instead of treating symptoms. Trends Pharmacol Sci 43(2):136–150 34895945 10.1016/j.tips.2021.11.004

[CR37] Pang Z, Lu Y, Zhou G, Hui F, Xu L, Viau C, Spigelman AF, MacDonald PE, Wishart DS, Li S, Xia J (2024) MetaboAnalyst 6.0: towards a unified platform for metabolomics data processing, analysis and interpretation. Nucleic Acids Res 52(W1):W398–W406 38587201 10.1093/nar/gkae253 PMC11223798

[CR38] Patitucci C, Couchy G, Bagattin A, Caneque T, de Reynies A, Scoazec JY, Rodriguez R, Pontoglio M, Zucman-Rossi J, Pende M, Panasyuk G (2017) Hepatocyte nuclear factor 1alpha suppresses steatosis-associated liver cancer by inhibiting PPARgamma transcription. J Clin Invest 127(5):1873–1888 28394260 10.1172/JCI90327 PMC5409083

[CR39] Pinzi L, Rastelli G (2019) Molecular docking: shifting paradigms in drug discovery. Int J Mol Sci. 10.3390/ijms20184331 31487867 PMC6769923

[CR40] Ponziani FR, Bhoori S, Castelli C, Putignani L, Rivoltini L, Del Chierico F, Sanguinetti M, Morelli D, Paroni Sterbini F, Petito V, Reddel S, Calvani R, Camisaschi C, Picca A, Tuccitto A, Gasbarrini A, Pompili M, Mazzaferro V (2019) Hepatocellular carcinoma is associated with gut microbiota profile and inflammation in nonalcoholic fatty liver disease. Hepatology 69(1):107–120 29665135 10.1002/hep.30036

[CR41] Sangro B et al (2025) EASL clinical practice guidelines on the management of hepatocellular carcinoma. J Hepatol 82(2):315–374 39690085 10.1016/j.jhep.2024.08.028

[CR42] Schwabe RF, Greten TF (2020) Gut microbiome in HCC - Mechanisms, diagnosis and therapy. J Hepatol 72(2):230–238 31954488 10.1016/j.jhep.2019.08.016

[CR43] Scott SA, Fu J, Chang PV (2020) Microbial tryptophan metabolites regulate gut barrier function via the aryl hydrocarbon receptor. Proc Natl Acad Sci U S A 117(32):19376–19387 32719140 10.1073/pnas.2000047117 PMC7431026

[CR44] Shannon P, Markiel A, Ozier O, Baliga NS, Wang JT, Ramage D, Amin N, Schwikowski B, Ideker T (2003) Cytoscape: a software environment for integrated models of biomolecular interaction networks. Genome Res 13(11):2498–2504 14597658 10.1101/gr.1239303 PMC403769

[CR45] Tilg H, Adolph TE, Trauner M (2022) Gut-liver axis: pathophysiological concepts and clinical implications. Cell Metab 34(11):1700–1718 36208625 10.1016/j.cmet.2022.09.017

[CR46] To JC, Chiu AP, Tschida BR, Lo LH, Chiu CH, Li XX, Kuka TP, Linden MA, Amin K, Chan WC, Bell JB, Moriarity BS, Largaespada DA, Keng VW (2021) ZBTB20 regulates WNT/CTNNB1 signalling pathway by suppressing PPARG during hepatocellular carcinoma tumourigenesis. JHEP Rep 3(2):100223 33604532 10.1016/j.jhepr.2020.100223 PMC7873381

[CR47] Tripathi A, Debelius J, Brenner DA, Karin M, Loomba R, Schnabl B, Knight R (2018) The gut-liver axis and the intersection with the microbiome. Nat Rev Gastroenterol Hepatol 15(7):397–411 29748586 10.1038/s41575-018-0011-z PMC6319369

[CR48] Wang R, Tang R, Li B, Ma X, Schnabl B, Tilg H (2021) Gut microbiome, liver immunology, and liver diseases. Cell Mol Immunol 18(1):4–17 33318628 10.1038/s41423-020-00592-6 PMC7852541

[CR49] Wang X, Zhang B, Jiang R (2025a) Microbiome interplays in the gut-liver axis: implications for liver cancer pathogenesis and therapeutic insights. Front Cell Infect Microbiol 15:1467197 39936163 10.3389/fcimb.2025.1467197 PMC11810975

[CR50] Wang Y, Peng X, Qian B, Wang L, Wang J (2025b) The integration of metabolites from *Forsythia suspensa* and gut microbiota ameliorates drug-induced liver injury: network pharmacology and molecular docking studies. Artif Cells Nanomed Biotechnol 53(1):105–121 40055878 10.1080/21691401.2025.2475088

[CR51] Wang Y, Wang X, Zhang X, Pan M, Sun M, Chen Z, Song Y (2025c) Utilizing network pharmacology and other tools to examine active components and mechanism of action of Magnolia officinalis rheum rhabarbarum decoction in treating Streptococcus pyogenes skin infections. Bioresour Bioprocess 12(1):92 40856949 10.1186/s40643-025-00933-1 PMC12381315

[CR52] Xia J, Ding H, Liu S, An R, Shi X, Chen M, Ren H (2023) C-type lectin receptors-triggered antifungal immunity may synergize with and optimize the effects of immunotherapy in hepatocellular carcinoma. J Inflamm Res 16:19–33 36636249 10.2147/JIR.S394503 PMC9831126

[CR53] Yao J, Ning B, Ding J (2025) The gut microbiota: an emerging modulator of drug resistance in hepatocellular carcinoma. Gut Microbes 17(1):2473504 40042184 10.1080/19490976.2025.2473504 PMC11901387

[CR54] Yao W, Huo J, Liu K, Tao P (2026) Unveiling the therapeutic potential of gut microbiota metabolites for the treatment of renal fibrosis based on network pharmacology study. Bioresour Bioprocess 13(1):94 42322381 10.1186/s40643-026-01083-8 PMC13283118

[CR55] Yeo YH, Abdelmalek M, Khan S, Moylan CA, Rodriquez L, Villanueva A, Yang JD (2025) Current and emerging strategies for the prevention of hepatocellular carcinoma. Nat Rev Gastroenterol Hepatol 22(3):173–190 39653784 10.1038/s41575-024-01021-z

[CR56] Yu J, Zhu P, Shi L, Gao N, Li Y, Shu C, Xu Y, Yu Y, He J, Guo D, Zhang X, Wang X, Shao S, Dong W, Wang Y, Zhang W, Zhang W, Chen WH, Chen X, Liu Z, Yang X, Zhang B (2024) *Bifidobacterium longum* promotes postoperative liver function recovery in patients with hepatocellular carcinoma. Cell Host Microbe 32(1):131-144 e136 38091982 10.1016/j.chom.2023.11.011

[CR57] Yu Q, Zhu L, Ding X, Lou Y (2025) Integration of network pharmacology and experimental validation to explore the pharmacological mechanism of andrographolide against asthma. Bioresour Bioprocess 12(1):30 40198539 10.1186/s40643-025-00869-6 PMC11979015

[CR58] Zhan X, Wu R, Kong XH, You Y, He K, Sun XY, Huang Y, Chen WX, Duan L (2023) Elevated neutrophil extracellular traps by HBV-mediated S100A9-TLR4/RAGE-ROS cascade facilitate the growth and metastasis of hepatocellular carcinoma. Cancer Commun (Lond) 43(2):225–245 36346061 10.1002/cac2.12388 PMC9926958

[CR59] Zhang X, Coker OO, Chu ES, Fu K, Lau HCH, Wang YX, Chan AWH, Wei H, Yang X, Sung JJY, Yu J (2021) Dietary cholesterol drives fatty liver-associated liver cancer by modulating gut microbiota and metabolites. Gut 70(4):761–774 32694178 10.1136/gutjnl-2019-319664 PMC7948195

[CR60] Zhao L, Zhang H, Li N, Chen J, Xu H, Wang Y, Liang Q (2023) Network pharmacology, a promising approach to reveal the pharmacology mechanism of Chinese medicine formula. J Ethnopharmacol 309:116306 36858276 10.1016/j.jep.2023.116306

