# Supplementary material for: Elucidating the beneficial effects of gut microbiota-derived metabolites in treating hepatocellular carcinoma: an integrated network pharmacology, molecular docking, and single-cell sequencing analysis
Source: Bioresour Bioprocess. 2026 Sep 12;13(1):133. doi: 10.1186/s40643-026-01129-x (PMC13570865; doi:10.1186/s40643-026-01129-x)
Supplement: Supplementary file 1 [file 40643_2026_1129_MOESM1_ESM.docx]

Fig. S1 Sensitivity analysis of PPI network using a more stringent confidence threshold (0.7).

Fig. S2 The chemical structures of the seven candidate metabolites.
