# Supplementary material for: Elucidating the beneficial effects of gut microbiota-derived metabolites in treating hepatocellular carcinoma: an integrated network pharmacology, molecular docking, and single-cell sequencing analysis
Source: Bioresour Bioprocess. 2026 Sep 12;13(1):133. doi: 10.1186/s40643-026-01129-x (PMC13570865; doi:10.1186/s40643-026-01129-x)
Supplement: Supplementary file 2 [file 40643_2026_1129_MOESM2_ESM.docx]

**Table S1. Gene name and degree of the common targets.**

| Gene name | Degree | Gene name | Degree | Gene name | Degree |
| --- | --- | --- | --- | --- | --- |
| AKT1 | 43 | MAPK1 | 23 | GCLC | 11 |
| IL6 | 42 | HDAC2 | 22 | HDAC7 | 11 |
| PPARG | 40 | HNF4A | 21 | ADRB2 | 9 |
| IL1B | 38 | MAPK14 | 21 | CTSD | 9 |
| JUN | 37 | CYP1A1 | 20 | HDAC11 | 8 |
| NFKB1 | 35 | NOS3 | 19 | CDK6 | 7 |
| CASP3 | 34 | HDAC4 | 18 | HDAC8 | 7 |
| PTGS2 | 32 | HDAC3 | 17 | LDHA | 7 |
| CXCL8 | 29 | NLRP3 | 16 | COL18A1 | 6 |
| FOS | 29 | NR1H4 | 16 | CYP2D6 | 6 |
| NFE2L2 | 29 | TLR9 | 15 | GPBAR1 | 6 |
| RELA | 27 | CYP3A4 | 14 | AHR | 3 |
| TLR4 | 27 | IDO1 | 14 | HPGD | 3 |
| HDAC1 | 26 | HDAC5 | 13 | RORA | 3 |
| CASP1 | 24 | HDAC9 | 12 | SSTR1 | 2 |
| GSK3B | 24 | NOX1 | 12 | SSTR3 | 2 |
| IL2 | 24 | CYP1A2 | 11 | IGFBP6 | 1 |
| MAPK8 | 24 | CYP27B1 | 11 |  |  |
| APP | 23 | G6PD | 11 |  |  |

**Table S2. Metabolite filtering cascade from gutMGene v2.0 to molecular docking.**

| Step | Description | Number of metabolites | Criteria / Action |
| --- | --- | --- | --- |
| 1 | Initial retrieval from gutMGene v2.0 | 277 | Homo sapiens |
| 2 | Target prediction (SEA + STP)  + HCC gene intersection | \ | protein‑coding genes + relevance score ≥ 1 (GeneCards) |
| 3 | PPI network construction  + hub gene identification | \ | topological algorithms → 8 core targets |
| 4 | Back‑mapping targets to gutMGene v2.0 | 31 | Associated with 8 core targets (Fig. 4) |
| 5 | Exclude "Unknown" entries | 10 named | 21 "Unknown" entries (Unknown4–Unknown24) excluded; 10 named metabolites retained |
| 6 | Exclude toxic metabolites | 3 | TMAO, hydroquinone, vancomycin excluded due to documented toxicity |
| 7 | Final candidates for docking and ADMET analysis | 7 | IPA, ketoA, genipin, succinate, acetate, propionate, butyrate |

**Table S3. Molecular docking results of candidate metabolites with core target proteins.**

| Protein(PDB ID) | Ligand | PubChem CID | Binding energy  (kcal/mol) | Grid box | | RMSD(Å) |
| --- | --- | --- | --- | --- | --- | --- |
|  |  |  |  | Center  (x, y, z) | Dimension  (x, y, z) |  |
| AKT1  (PDB ID:3MV5) | Co-crystal ligand | — | -7.6 | (9, -2, 21) | (19, 34, 19) | 0.00 |
|  | IPA | 3744 | -7.1 | (9, -2, 21) | (19, 34, 19) | - |
|  | Acetate | 175 | -3.3 | (12, 10, 36) | (14, 14, 14) | - |
|  | Propionate | 104745 | -3.7 | (12, 10, 36) | (15, 15, 15) | - |
|  | Butyrate | 104775 | -4.2 | (12, 10, 36) | (15, 15, 15) | - |
|  | Succinate | 160419 | -4.6 | (12, 10, 36) | (17, 17, 17) | - |
|  | Genipin | 442424 | -6.5 | (9, -2, 21) | (18, 34, 18) | - |
|  | KetoA | 24970825 | -6.1 | (9, -2, 21) | (26, 34, 26) | - |
| IL6  (PDBID:1A1U) | Co-crystal ligand | — | -4.7 | (14, -31, 0) | (16, 16, 16) | 1.157 |
|  | IPA | 3744 | -6.0 | (14, -31, 0) | (19, 19, 19) | - |
|  | Acetate | 175 | -3.0 | (14, -31, 0) | (14, 14, 14) | - |
|  | Propionate | 104745 | -3.4 | (14, -31, 0) | (15, 15, 15) | - |
|  | Butyrate | 104775 | -3.7 | (14, -31, 0) | (15, 15, 15) | - |
|  | Succinate | 160419 | -4.3 | (14, -31, 0) | (17, 17, 17) | - |
|  | Genipin | 442424 | -5.3 | (14, -31, 0) | (18, 18, 18) | - |
|  | KetoA | 24970825 | -5.0 | (6, -24, 18) | (26, 26, 26) | - |
| PPARG  (PDB ID:6TSG) | Co-crystal ligand | — | -6.6 | (-6, -5, -38) | (22, 22, 22) | 1.909 |
|  | IPA | 3744 | -6.6 | (-3, 0, -16) | (30, 25, 28) | - |
|  | Acetate | 175 | -3.1 | (-17, 21, -36) | (14, 14, 14) | - |
|  | Propionate | 104745 | -3.6 | (-17, 21, -36) | (15, 15, 15) | - |
|  | Butyrate | 104775 | -4.1 | (-6, -5, -38) | (15, 15, 15) | - |
|  | Succinate | 160419 | -4.3 | (-3, 0, -16) | (30, 25, 28) | - |
|  | Genipin | 442424 | -6.4 | (-3, 0, -16) | (30, 25, 28) | - |
|  | KetoA | 24970825 | -6.4 | (-3, 0, -16) | (26, 26, 26) | - |
| JUN  (PDB ID:6Y3V) | - | - | - | - | - | - |
|  | IPA | 3744 | -5.3 | (33, -26, 2) | (19, 19, 19) | - |
|  | Acetate | 175 | -2.9 | (33, -26, 2) | (21, 14, 14) | - |
|  | Propionate | 104745 | -3.4 | (33, -26, 2) | (21, 15, 15) | - |
|  | Butyrate | 104775 | -3.7 | (33, -26, 2) | (21, 15, 15) | - |
|  | Succinate | 160419 | -4.1 | (29, -3, -22) | (17, 17, 17) | - |
|  | Genipin | 442424 | -5.0 | (21, -21, 3) | (18, 18, 18) | - |
|  | KetoA | 24970825 | -5.1 | (21, -21, 3) | (26, 26, 26) | - |
| IL1B  (PDB ID:1HIB) | - | - | - | - | - | - |
|  | IPA | 3744 | -5.6 | (19, 3, 84) | (19, 19, 19) | - |
|  | Acetate | 175 | -3.2 | (35, 6, 69) | (14, 14, 14) | - |
|  | Propionate | 104745 | -3.6 | (35, 6, 69) | (15, 15, 15) | - |
|  | Butyrate | 104775 | -3.8 | (35, 6, 69) | (15, 15, 15) | - |
|  | Succinate | 160419 | -4.9 | (35, 6, 69) | (17, 17, 17) | - |
|  | Genipin | 442424 | -5.4 | (18, -9, 68) | (18, 18, 18) | - |
|  | KetoA | 24970825 | -4.8 | (19, 3, 84) | (26, 26, 26) | - |
| NFKB1  (PDB ID:8TQD） | Co-crystal ligand | - | -12.1 | (9, -2, -13) | (20, 20, 20) | 0.000 |
|  | IPA | 3744 | -5.7 | (21, 3, 13) | (19, 19, 19) | - |
|  | Acetate | 175 | -3.1 | (21, 9, -7) | (14, 14, 14) | - |
|  | Propionate | 104745 | -3.7 | (21, 9, -7) | (15, 15, 15) | - |
|  | Butyrate | 104775 | -4.1 | (21, 9, -7) | (15, 15, 15) | - |
|  | Succinate | 160419 | -4.5 | (21, 3, 13) | (17, 17, 17) | - |
|  | Genipin | 442424 | -5.4 | (21, 3, 13) | (18, 18, 18) | - |
|  | KetoA | 24970825 | -5.0 | (21, 3, 13) | (26, 26, 26) | - |

**Continue Table S3. Molecular docking results of candidate metabolites with core target proteins (continued)**

| Protein(PDB ID) | Ligand | PubChem CID | Binding energy  (kcal/mol) | Grid box | | RMSD (Å) |
| --- | --- | --- | --- | --- | --- | --- |
|  |  |  |  | Center  (x, y, z) | Dimension  (x, y, z) |  |
| CASP3  (PDB ID:6BDV) | Co-crystal ligand | - | -3.6 | (7, 32, 98) | (13, 13, 13) | 0.000 |
|  | IPA | 3744 | -5.5 | (0, 38, 112) | (19, 19, 19) | - |
|  | Acetate | 175 | -2.6 | (7, 32, 98) | (14, 14, 14) | - |
|  | Propionate | 104745 | -3.0 | (7, 32, 98) | (15, 15, 15) | - |
|  | Butyrate | 104775 | -3.3 | (7, 32, 98) | (15, 15, 15) | - |
|  | Succinate | 160419 | -3.6 | (7, 32, 98) | (17, 17, 17) | - |
|  | Genipin | 442424 | -5.2 | (0, 38, 112) | (18, 18, 18) | - |
|  | KetoA | 24970825 | -4.7 | (0, 38, 112) | (26, 26, 26) | - |
| PTGS2  (PDB ID:3HS5） | Co-crystal ligand | - | -6.4 | (30, 38, 28) | (19, 29, 34) | 0.743 |
|  | IPA | 3744 | -7.4 | (14, 14, 71) | (29, 26, 28) | **-** |
|  | Acetate | 175 | -3.1 | (25, 28, 43) | (35, 35, 35) | - |
|  | Propionate | 104745 | -3.6 | (25, 28, 43) | (35, 35, 35) | - |
|  | Butyrate | 104775 | -4.2 | (30, 38, 28) | (24, 29, 34) | - |
|  | Succinate | 160419 | -4.8 | (30, 38, 28) | (24, 29, 34) | - |
|  | Genipin | 442424 | -7.4 | (30, 38, 28) | (24, 29, 34) | - |
|  | KetoA | 24970825 | -6.6 | (11, 19, 20) | (26, 26, 26) | - |

**Note:** RMSD‑based re‑docking validation was not applicable for JUN (6Y3V) and IL-1B (1HIB), as these structures lack co‑crystallized small‑molecule ligands (6Y3V is a protein–peptide complex; 1HIB is a protein‑only mutant).

**Table S4. The details of the molecular docking test for re-docking validation**

| Protein(PDB ID) | Ligand | PubChem CID | Binding energy  (kcal/mol) | Grid box | |
| --- | --- | --- | --- | --- | --- |
|  |  |  |  | Center  (x, y, z) | Dimension  (x, y, z) |
| AKT1  (PDBID:7NH5) | IPA | 3744 | -7.1 | (10, -14, 12) | (19, 34, 19) |
|  | Acetate | 175 | -3.3 | (10, -14, 12) | (14, 14, 14) |
|  | Propionate | 104745 | -3.7 | (10, -14, 12) | (15, 15, 15) |
|  | Butyrate | 104775 | -4.2 | (10, -14, 12) | (15, 15, 15) |
|  | Succinate | 160419 | -4.6 | (10, -14, 12) | (17, 17, 17) |
|  | Genipin | 442424 | -6.5 | (10, -14, 12) | (18, 34, 18) |
|  | KetoA | 24970825 | -6.1 | (10, -14, 12) | (26, 34, 26) |
| PPARG  (PDB ID:4XLD) | IPA | 3744 | -6.6 | (55, 27, 2) | (30, 25, 28) |
|  | Acetate | 175 | -3.1 | (55, 27, 2) | (14, 14, 14) |
|  | Propionate | 104745 | -3.6 | (55, 27, 2) | (15, 15, 15) |
|  | Butyrate | 104775 | -4.1 | (55, 27, 2) | (15, 15, 15) |
|  | Succinate | 160419 | -4.3 | (55, 27, 2) | (30, 25, 28) |
|  | Genipin | 442424 | -6.4 | (55, 27, 2) | (30, 25, 28) |
|  | KetoA | 24970825 | -6.4 | (55, 27, 2) | (26, 26, 26) |
| CASP3  (PDB ID:2CNL) | IPA | 3744 | -5.5 | (22, 7, 35) | (19, 19, 19) |
|  | Acetate | 175 | -2.6 | (18, 13, 52) | (14, 14, 14) |
|  | Propionate | 104745 | -3.0 | (27, 49, 47) | (15, 15, 15) |
|  | Butyrate | 104775 | -3.3 | (27, 49, 47) | (15, 15, 15) |
|  | Succinate | 160419 | -3.6 | (49, 23, 36) | (17, 17, 17) |
|  | Genipin | 442424 | -5.2 | (22, 7, 35) | (18, 34, 18) |
|  | KetoA | 24970825 | -4.7 | (22, 7, 35) | (26, 26, 26) |

**Notes**:Positive control re-docking was performed using the co-crystallized native ligand of each protein: AKT1 (PDB:

7NH5, co‑crystallized with a covalent‑allosteric inhibitor) , PPARG (PDB: 4XLD, co‑crystallized with rosiglitazone) ,

and CASP3 (PDB: 2CNL, co‑crystallized with an aza‑peptide epoxide inhibitor) .
